# Transcriptional regulation of Hepatic Stellate Cell activation in NASH

**DOI:** 10.1038/s41598-019-39112-6

**Published:** 2019-02-20

**Authors:** Ann-Britt Marcher, Sofie M. Bendixen, Mike K. Terkelsen, Sonja S. Hohmann, Maria H. Hansen, Bjørk D. Larsen, Susanne Mandrup, Henrik Dimke, Sönke Detlefsen, Kim Ravnskjaer

**Affiliations:** 1 Department of Biochemistry and Molecular Biology, University of Southern Denmark, 5230 Odense M, Denmark; 2 Department of Cardiovascular and Renal Research, Institute of Molecular Medicine, University of Southern Denmark, 5000 Odense C, Denmark; 3 Department of Pathology, Odense University Hospital, 5000 Odense C, Denmark

## Abstract

Non-alcoholic steatohepatitis (NASH) signified by hepatic steatosis, inflammation, hepatocellular injury, and fibrosis is a growing cause of chronic liver disease, cirrhosis, and hepatocellular carcinoma. Hepatic fibrosis resulting from accumulation of extracellular matrix proteins secreted by hepatic myofibroblasts plays an important role in disease progression. Activated hepatic stellate cells (HSCs) have been identified as the primary source of myofibroblasts in animal models of hepatotoxic liver injury; however, so far HSC activation and plasticity have not been thoroughly investigated in the context of NASH-related fibrogenesis. Here we have determined the time-resolved changes in the HSC transcriptome during development of Western diet- and fructose-induced NASH in mice, a NASH model recapitulating human disease. Intriguingly, HSC transcriptional dynamics are highly similar across disease models pointing to HSC activation as a point of convergence in the development of fibrotic liver disease. Bioinformatic interrogation of the promoter sequences of activated genes combined with loss-of-function experiments indicates that the transcriptional regulators ETS1 and RUNX1 act as drivers of NASH-associated HSC plasticity. Taken together, our results implicate HSC activation and transcriptional plasticity as key aspects of NASH pathophysiology.

## Introduction

Obesity, insulin resistance, and type-2 diabetes drive an epidemic of non-alcoholic fatty liver disease (NAFLD)^1–3^. NAFLD has a global prevalence of 25% and its progressive form, non-alcoholic steatohepatitis (NASH), is now the most common cause of chronic liver disease^3^. Histologically, NASH is characterized by hepatic lipid accumulation, intralobular inflammation, and fibrosis^4^. Recent studies identify even early-stage hepatic fibrosis as an independent predictor of both overall and liver-related mortality for NAFLD patients^5–9^. Functional insight into the mechanisms underlying NASH, hepatic fibrogenesis, and extracellular matrix (ECM) turnover is therefore critical to the development of feasible treatment strategies and mortality reduction.

Fate-tracing experiments in mice have identified activated hepatic stellate cells (HSCs) as precursors for ECM-producing myofibroblasts in mice treated with carbon tetrachloride (CCl_4_), fed a methionine/choline-deficient (MCD) diet, or subjected to bile duct ligation^10^. Quiescent HSCs represent 5–10% of cells in the healthy liver and are activated upon autocrine and paracrine stimulation with growth factors and cytokines secreted from resident and infiltrating cells. Experimentally established inducers of fibrogenesis include TGFβ^11^, PDGFβ^12^, and CTGF^13^ signaling through their cognate receptors and integrins. Integrins also promote HSC activation by facilitating growth factor activation^14^ and as receptors for ECM components in mechanotransduction^15^. Upon receptor activation, signals are transduced by interlinked FAK-RHO, RAC and MAP-kinase pathways (reviewed in)^16,17^.

While it is recognized that activation and transdifferentiation of quiescent HSCs to myofibroblasts involves profound changes in gene expression, little is known about the transcriptional effectors of the above signals. The best described transcriptional regulators of HSC transdifferentiation are the transcription factors (TFs) SMAD3 and STAT3 conveying growth factor and cytokine signals to the genome^18,19^, but other transcriptional regulators, including YAP1^20^, GLI2^21^, AP-1^22^, SOX9^23^, and ETS family members^24,25^, may also be involved by activating key fibrogenic genes.

Several studies comparing *global* gene expression in quiescent and activated HSCs have been published in recent years^26–32^. Human or murine HSCs were activated *in vitro* or isolated from mice either treated with CCl_4_^29,30^, fed an MCD diet^29^, or infected with *Schistosoma japonicum*^31^. Currently, there are no reports firmly linking HSC activation to NASH-associated fibrogenesis and no data available on HSC gene expression in human NASH or pathophysiologically equivalent models of human NASH. Although an increasing number of studies include *global* analyses of hepatic gene expression in NAFLD and NASH^33–36^, none of these offer the cell type resolution to address NASH-associated HSC plasticity or the transcriptional basis for HSC activation.

By time-resolved gene expression profiling of isolated HSCs we here determine the transcriptional programs that define early HSC activation in diet-induced NASH in mice. By comparing with established models of HSC activation we show highly similar transcriptional dynamics in HSCs across models of *in vivo* activation and identify ETS1 and RUNX1 TF motifs as highly significant predictors of HSC gene induction in NASH and early fibrosis. Accordingly, we show that acute loss of ETS1 and RUNX1 function attenuates HSC activation.

## Results

### Hepatic stellate cell activation and induction of fibrosis by Western diet and fructose feeding

**Figure 1:**
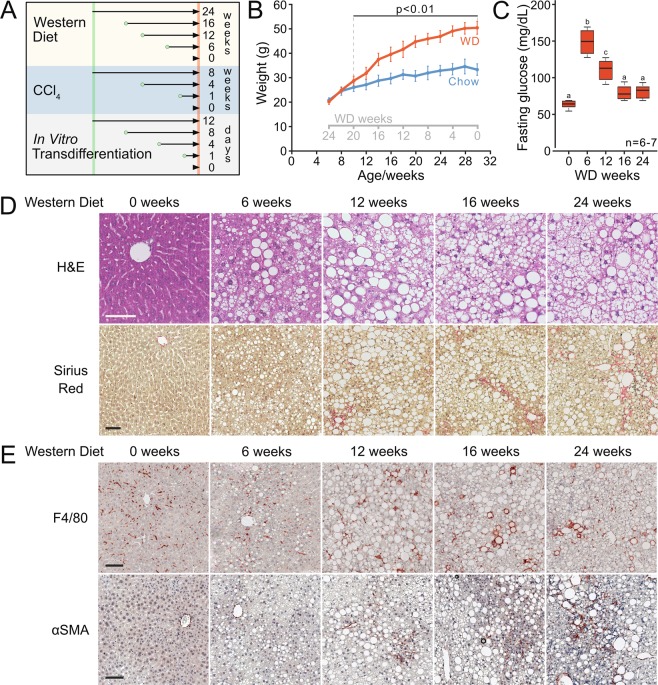
Western diet and fructose-fed mice recapitulate features of human NASH. (**A**) Experimental outline showing time courses for Western diet (WD)-feeding and CCl_4_-treatment of mice, and *in vitro* transdifferentiation of isolated HSCs. (**B**,**C**) Body weights (WD vs. chow; p <0.01) and fasting (16 hours) blood glucose of WD-fed mice (n =6–7). Means not sharing a letter are significantly different (p_adj_ <0.05, ANOVA, Tukey-adjusted). (**D**) Hematoxylin and Eosin (H&E) and Sirius red staining of FFPE liver sections from WD- or chow-fed mice (scale bars 100 μm). (**E**) F4/80^+^ and αSMA^+^ IHC on FFPE liver sections from WD- or chow-fed mice (scale bars 100 μm).

For diet-induced HSC activation, male C57BL/6J mice were fed a Western diet (Supplementary Table T1) supplemented with 42 g/L D-fructose (WD) in their drinking water for 6, 12, 16, or 24 weeks. Control mice were fed normal chow and pure drinking water. To compare mice of the same chronological age, WD feeding was initiated at 6, 14, 18, and 24 weeks of age, respectively, and continued until 30 weeks of age (Fig. 1A). At 30 weeks of age, mean body weights were 33.2 ± 2.4 g and 50.6 ± 2.6 g for chow-fed and 24-week WD-fed mice, respectively (Fig. 1B). Fasting blood glucose was initially significantly elevated by WD with the highest levels after six weeks (Fig. 1C) and later normalized. The biphasic trend in fasting glycemia likely reflects the onset of insulin resistance and subsequent compensation by the endocrine pancreas^37^. Age-matched, chow-fed mice showed no change in fasting blood glucose during the study.

To compare our WD model to established models of HSC activation we treated separate cohorts of male C57BL/6J mice with CCl_4_, a known inducer of HSC activation and hepatic fibrosis in rodents^38^. CCl_4_ was given twice weekly for 1, 4, or 8 weeks, whereas control mice received vehicle for 8 weeks. Activation of HSCs was confirmed by the appearance of centrilobular αSMA expression over the 8 weeks of CCl_4_ treatment (Supplementary Fig. 1A). As a third, independent model, we induced activation and transdifferentiation of HSCs from healthy C57BL/6J mice to myofibroblasts *in vitro* (*IVT*). HSC transdifferentiation was achieved after 12 days in culture as assessed by αSMA immunofluorescence (IF) and phase contrast microscopy (Supplementary Fig. 1B,C). Representative FACS plots from our sorting of isolated HSCs are shown in Supplementary Fig. 1D.

Hepatic steatosis was prominent from 6 weeks of WD and increased over time, reaching steatosis score 3 in all but one case (Fig. 1D). Hepatocytes exhibit mainly macrovesicular steatosis, characterized by large lipid droplets and peripheral nuclei. As a morphological feature of NASH, inflammatory foci are observed within the steatotic lobules. In most cases, one inflammatory focus was observed per medium-power field. From 24 weeks of WD, localized, pericellular fibrosis is seen, mainly in zone 3, corresponding to Kleiner fibrosis stage I (Fig. 1D). These histological findings were recapitulated by label-free CARS/SHG microscopy (Supplementary Fig. 1E). Bridging fibrosis is observed in livers from CCl_4_-treated mice (Supplementary Fig. 1F).

Since WD led to intralobular inflammation and fibrosis, we assessed hepatic content of F4/80-positive (F4/80^+^) macrophages as well as smooth muscle actin-positive (αSMA^+^) myofibroblasts. Already after 6 weeks, redistribution of F4/80^+^ cells was seen, paralleling the overall change in tissue architecture (Fig. 1E). Coinciding with aggravated steatosis and steatohepatitis, F4/80^+^ cells formed crown-like structures (CLSs) around lipid-laden hepatocytes by WD week 12. CLS formation has previously been shown to correlate with hepatocellular injury in human NASH^39^ but were not observed in livers from CCl_4_-treated mice (Supplementary Fig. 1F). From WD week 12, discrete patches with αSMA^+^ cells became visible, likely reflecting the emergence of myofibroblasts (Fig. 1E)^40^.

Collectively, we show that WD and fructose feeding of C57BL/6J mice is a relevant model of human NASH with steatosis, crown-like structures and αSMA^+^ cells emerging after 12 weeks, and low-grade, pericellular fibrosis after 24 weeks.

### HSC transcriptional plasticity in Western diet and fructose-fed mice

Finding that WD induced hepatic αSMA expression and fibrosis, we wanted to examine the transcriptional plasticity of HSCs during WD-induced NASH development. We therefore isolated and FACS-sorted HSCs from WD-fed mice for gene expression profiling over the course of our experiment. Guided by our histopathology findings, we sequenced HSC mRNA from mice fed WD for 12, 16, or 24 weeks and from chow-fed control mice (WD, 0w) (n =4). In parallel, we sequenced mRNA from isolated HSCs from mice treated with CCl_4_ for 1, 4, or 8 weeks or vehicle (ctrl) (n =3–4) as well as from HSCs activated *in vitro* for 1, 4, 8, or 12 days (n =3). HSCs allowed to recover in suspension for two hours post isolation were used as *IVT* controls *(IVT*, d0).

**Figure 2:**
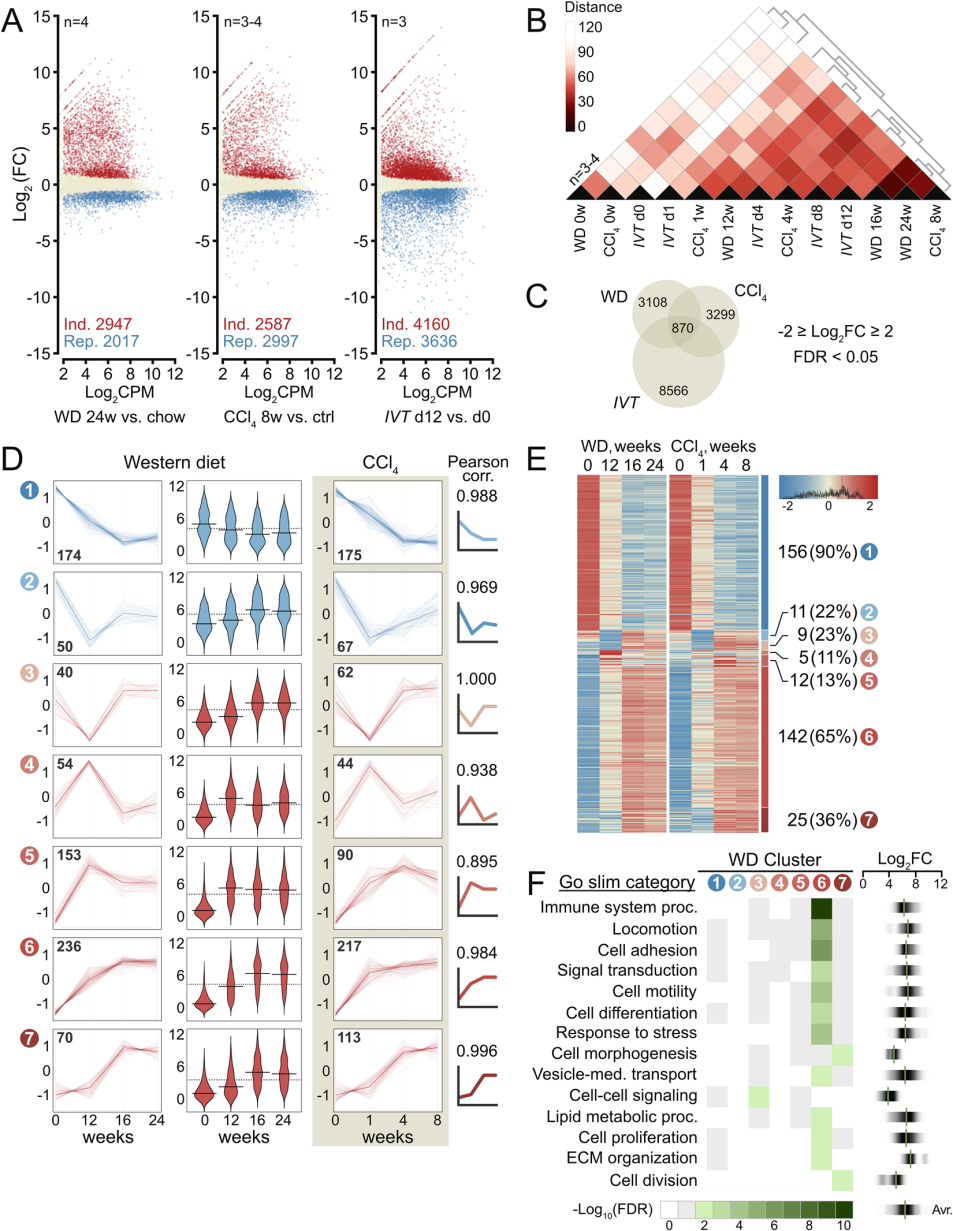
Time-resolved transcriptomic analysis of HSC activation. (**A**) Log_2_FC for all expressed genes in WD, CCl_4_, and *IVT* experiments relative to controls (log_2_CPM >2). Induced and repressed genes (FDR ≤0.05), shown in red and blue, respectively. Gene numbers are indicated. (**B**) Distance plot and k-means clustering of all samples of the three experiments based on genes differentially expressed (FDR ≤0.01) between HSCs from 24w WD- and chow-fed mice. (**C**) 870 *common* genes induced ≥4-fold (FDR <0.05), between any two time points in all three experiments, or repressed ≥4-fold (FDR  <0.05). (**D**) *Common* genes clustered by profiles of variance-stabilized, scaled exon counts through WD and CCl_4_ experiments. Gene counts and mean aggregate profiles are shown (bold line). Pearson’s correlation coefficient of mean aggregate profiles are shown for cluster pairs. Middle panel bean plots show normalized log_2_(RPKM) values and medians for each WD cluster. Dashed lines show average log_2_(RPKM) values across time course. (RPKM: Reads Per Kilobase of transcript per Million mapped reads). (**E**) Exon counts for 360 genes in WD clusters 1–7, which are *shared* with corresponding CCl_4_ clusters. Number of *shared* genes in each cluster pair, percent of parent WD cluster, and cluster numbers (circles) are shown. (**F**) Enriched (green; FDR ≤0.01) go-slim categories and -log_10_(FDR) values for each WD cluster. *Common* genes expressed at RPKM ≥10 at any time point in the three experiments are included in the go-analysis. Density strip plots show distributions and median log_2_FC values (24w WD vs. chow) for genes of enriched go-slim categories within respective clusters. All values are averages of 3–4 biological replicates.

Analysis of differentially expressed genes revealed profound differences between WD-activated and quiescent HSCs. A total of 2947 genes were significantly induced (FDR ≤0.05; log_2_CPM >2) and 2017 genes significantly repressed by WD for 24 weeks (Fig. 2A). Similarly, CCl_4_ treatment and *in vitro* activation induced 2587 and 4160 HSC genes, respectively, while 2997 and 3636 genes were repressed. Activated HSCs in all three models show shifts in gene expression distributions towards higher overall transcriptional activity (p <1E-15; Supplementary Fig. 2A). Importantly, K-means and hierarchical clustering of samples across models show highly similar changes in HSC gene expression in mice fed WD for 16 or 24 weeks and mice given CCl_4_ for 8 weeks (Fig. 2B and Supplementary Fig. 2B,D). This similarity is recapitulated in the principal component analysis where WD- and CCl_4_-samples align closely and where PC2 captures activation-associated changes common across all three models (Supplementary Fig. 2C). Expression of key HSC activation markers for all three models are shown in Supplementary Fig. 2E.

To focus on functionally significant changes in HSC gene regulation upon WD-feeding we identified co-regulated genes across our three models with similar *topographies* during HSC-activation. First, we extracted genes robustly regulated across our experimental models. A total of 870 *common* genes were activated at least four-fold in all three models or were four-fold repressed (Fig. 2C; FDR <0.05). We then subjected these 870 genes to unsupervised time-course clustering^41^ entering variance-stabilized, scaled expression values from the WD time course. PAMK clustering by Pearson’s correlation distance metrics returned seven clusters of genes with highly congruent expression profiles over the course of WD feeding (Fig. 2D; avr. pearson =0.968). The 10% least fitting genes and a single cluster containing only six genes were discarded during the analysis. The number of genes in each cluster is indicated and the mean aggregate profile for each cluster is shown in bold. Bean plots show log_2_-transformed RPKM values for genes in each WD cluster.

We next reran the clustering algorithm using HSC gene expression values from our CCl_4_ time course (Fig. 2D). Remarkably, the resulting seven gene clusters show topographies highly similar to the WD-clusters despite the distinct time courses and profoundly different histopathology of the two models. We paired WD and CCl_4_ clusters based on prototype similarities (Pearson’s correlation; Fig. 2D, right) and extracted 360 *shared* genes present in both WD and CCl_4_ clusters of the resulting pairs (Fig. 2E). Two cluster pairs in particular, 1 and 6, show highly significant overlaps with 156 (90% of WD cluster 1) and 142 (65% of WD cluster 6) shared genes, respectively (see Supplementary Table T2 for hypergeometric probabilities and T5 for list of genes).

The striking similarity in temporal gene expression profiles across models of HSC activation indicates the existence of core transcriptional programs and prompted us to perform functional enrichment analyses of each cluster of WD-regulated genes. To include only robustly expressed genes in the analyses we applied a threshold of RPKM ≥10 at any time point in our three models. Gene ontology (GO) analysis of each WD cluster revealed 14 enriched GO-Slim categories (FDR ≤0.01), 11 of which are enriched for genes in cluster 6 (Fig. 2F). Among these categories are *immune system process*, *cell adhesion*, and *ECM organization*, which recapitulate findings from human NASH and independent murine NASH models^33,35,36^. Other categories; *locomotion*, *cell differentiation*, and *cell proliferation* specifically reflect HSC activation and were not identified in whole liver datasets. Density strip plots show median log_2_ fold changes (FC) at WD 24w vs. chow of genes in each GO-Slim category within the respective cluster. Notably, no categories are enriched among the repressed genes of the highly conserved WD cluster 1. Median log_2_FC values across all enriched GO-Slim categories therefore average to 6.28 in keeping with the overall increase in transcriptional activity upon HSC activation in NASH.

We here show the profound transcriptional plasticity in HSCs during murine NASH development. By time-resolved transcriptomic analyses of HSCs through disease development we have discovered the orchestrated induction of a core myofibroblastic program remarkably conserved across models of HSC activation.

### ETS1 and RUNX1 are putative fibrogenic drivers in NASH

**Figure 3:**
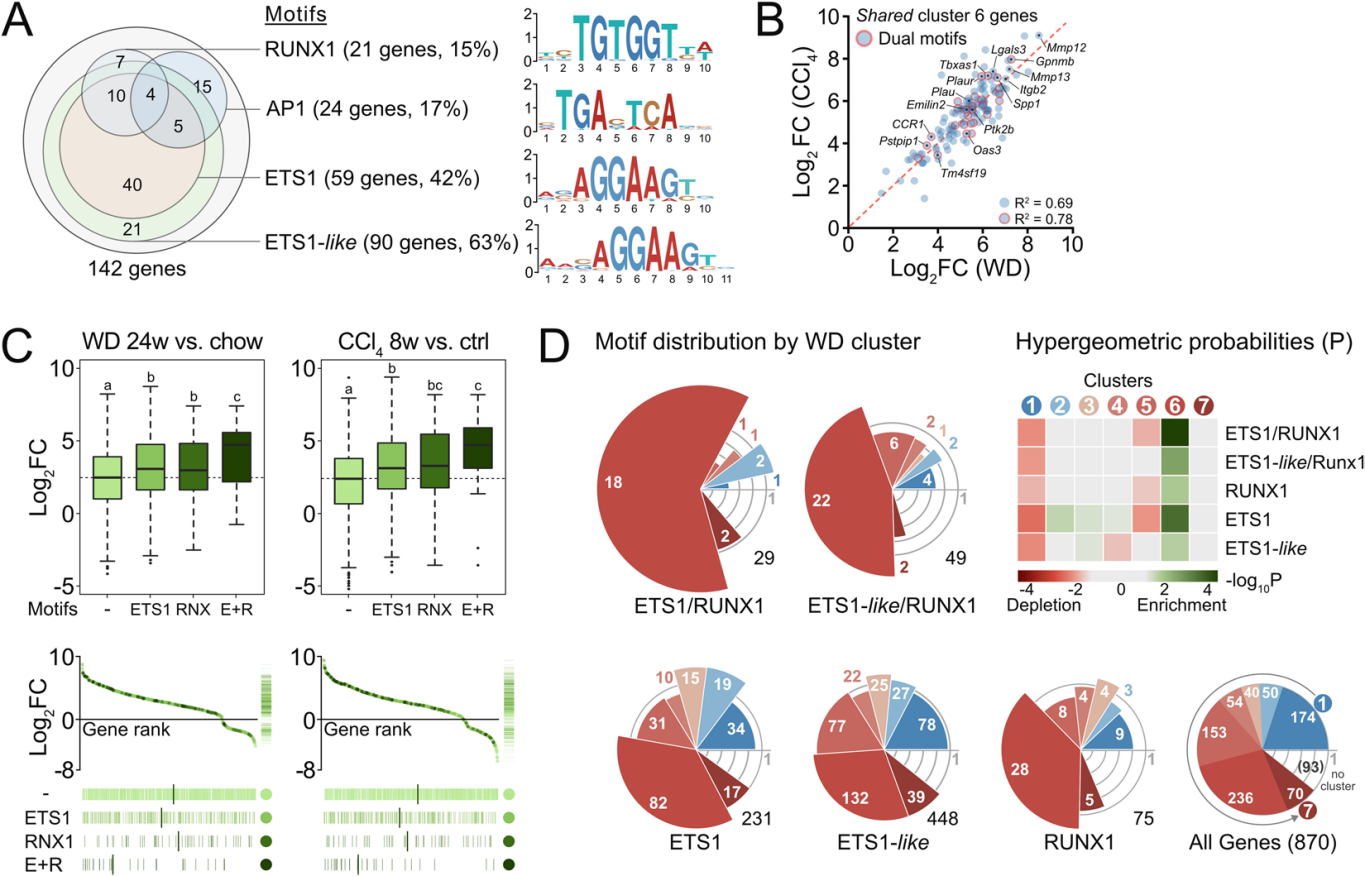
ETS1 and RUNX1 motifs predict HSC gene induction in NASH. (**A**) Proximal promoters (−300; +50) of *shared* genes in WD and CCl_4_ clusters 6 with putative RUNX1, AP-1, ETS1 and ETS1-*like* sites. Motif logos generated from putative binding sites in corresponding promoters. (**B**) Correlation of log_2_FC values for *shared* genes of WD and CCl_4_ clusters 6 with indication of genes with dual ETS1/ETS1-like and RUNX1 or AP-1 motifs. (**C**) Log_2_FC in expression of the 870 *common* genes in WD and CCl_4_ experiments grouped by the presence of putative ETS1 (231 genes) and RUNX1 (Rnx; 75 genes) binding sites or both (E + R; 29 genes). Dashed horizontal lines indicate average log_2_FC for all *common* genes in experiment. Means not sharing a letter are significantly different (FDR <0.05, pairwise Wilcoxon rank-sum test, Benjamini-Hochberg corrected). Lower panel: Rug plots showing genes ranked by log_2_FC and shaded by motif. Median rank shown for each motif group. (**D**) Distribution of genes with ETS1, ETS1-*like*, and RUNX1 motifs over WD clusters. Slices color-coded by WD cluster and showing number of genes with motifs. Slice areas scaled by enrichment of motif-containing genes in respective cluster over all *common* genes with motifs (numbers in black). Heat map shows hypergeometric probabilities (P) of the distribution of genes with motifs across clusters. “No cluster” refers to 10% least fitting *common* genes. All values are averages of 3–4 biological replicates.

Having found clusters of genes linked to key pathophysiological processes and co-regulated across models of HSC activation, we set out to identify central transcriptional regulators. We used motif finding algorithms^42^ to discover enriched TF binding motifs within promoters of the *shared* genes in our cluster pairs. Significantly enriched TF binding motifs were found only in promoters of the 142 *shared* genes of WD and CCl_4_ cluster 6 (Supplementary Table T3). Of 19 motifs, 12 are ETS TF family motifs (ETS1-*like*), one is the RUNX1 motif, and six are bZIP-TF (e.g. AP-1) motifs. Notably, two-thirds (90) of the 142 promoters have one or more ETS1-*like* motif, 21 promoters have RUNX1 motifs, and 24 have bZip/AP1 motifs (Fig. 3A). Motif logos generated from the actual promoter sequences are shown for top motifs. While all ETS1-*like* motifs are canonical *in vivo* motifs of the ETS family Class-I TFs^43^, 59 were specifically identified as cognate ETS1 sites. The significance of this distinction, however, is unclear given the known redundancy of ETS family TF binding in promoter regions^44^. Comparing induction of the *shared* cluster 6 genes from WD and CCl_4_ model HSCs, we find that genes with dual ETS1-*like*/RUNX1 or ETS1-*like*/AP-1 motifs show a particularly strong correlation between models (R^2^ =0.78; Fig. 3B). This further strengthens the notion that cluster 6 genes constitute a conserved transcriptional program in activated HSCs *in vivo* and points to the ETS family TFs, RUNX1, and AP-1 dimers as putative regulators hereof.

For each HSC activation model, we also compiled all genes with increased or reduced expression between any two time points and performed motif enrichment analysis either of all the genes or the genes induced or repressed exclusively in that model and not the others (Supplementary Fig. 3A). Specifically, in genes induced in WD-fed mice we see motifs for hepatocyte nuclear factors HNF1A, HNF1B, and HNF4A as well as the peroxisome proliferator-activated receptor alpha (PPARA); TFs usually not associated with HSCs. WD-feeding led to massive steatosis and we speculate that mRNA from disrupted hepatocytes and co-fractionation of triglyceride-rich debris with our HSCs may have resulted in increased numbers of hepatocyte-derived transcripts. Indeed, also transcript levels for these hepatocyte-specific TFs are increased in sequencing libraries from WD-fed mice but still very low relative to levels in whole liver (Supplementary Fig. 3B). By comparison, HSC transcripts of Desmin (*Des*), Lecithin-retinol acyltransferase (*Lrat*), Retinol-binding protein 1 (*Rbp1*), and Vimentin (*Vim*) are highly enriched relative to whole liver. While the broader enrichment analysis gives a wide array of motifs for further validation, our stringent analysis of *common* genes guided by temporal expression profiles identifies core motifs shared across all three models and reduces experimental noise. A heat map with hierarchical clustering of the enriched motifs based on their similarities (Pearson’s correlation) is shown in Supplementary Fig. 3C.

By grouping the *common* 870 genes regulated ≥4-fold in all three models (see Fig. 2C) by the presence of either motif or their combinations we find a strong correlation between these motifs and gene induction. Genes with either ETS1 or RUNX1 (RNX) motifs in their proximal promoters were significantly more induced in HSCs upon *in vivo* activation by WD or CCl_4_ than genes without (Fig. 3C). In particular, genes having both ETS1 and RUNX1 (E + R) motifs show a median log_2_FC of 4.72 (WD 24w vs. chow) compared to 2.50 for genes without these motifs. These gene subsets show median log_2_FCs of 4.69 and 2.40, respectively, in HSCs from CCl_4_-treated mice suggestive of a general mechanism across models. Correspondingly, individual genes with both motifs rank considerably higher in inducibility than genes with either motif alone or no motif (Fig. 3C). A significant effect of the AP-1 motif but no motif cooperativity is observed for regulated genes with dual ETS1 and AP-1 motifs in our *in vivo* models (median log_2_FC =3.41 (WD) and 3.49 (CCl_4_); Supplementary Fig. 4A) prompting us to focus on the ETS1-RUNX1 cooperativity.

A closer look at motif distributions across WD gene clusters reveals a strong enrichment of genes with dual ETS1/ETS1-*like* and RUNX1 motifs in the induced WD cluster 6 and a significant depletion in the repressed cluster 1 (Fig. 3D). Of the 29 ETS1 and RUNX1 dual motif-containing genes, 18 belong to WD cluster 6. Genes with individual motifs exhibit a similar cluster-distribution indicating a causal role of these motifs and their combinations in defining HSC gene expression profiles in NASH. In support of such functional roles for ETS1 and RUNX1 in HSC activation, enrichment of ETS1 and RUNX motifs is replicated for genes falling into the previously identified Go-Slim categories (Supplementary Fig. 4B). Of note, induction of Runx1 itself (log_2_FC =3.0; WD 24w vs. chow and log_2_FC =4.1; CCl_4_ 8w vs. ctrl) may contribute to a sustained expression of RUNX1 motif-containing genes beyond HSC activation.

**Figure 4:**
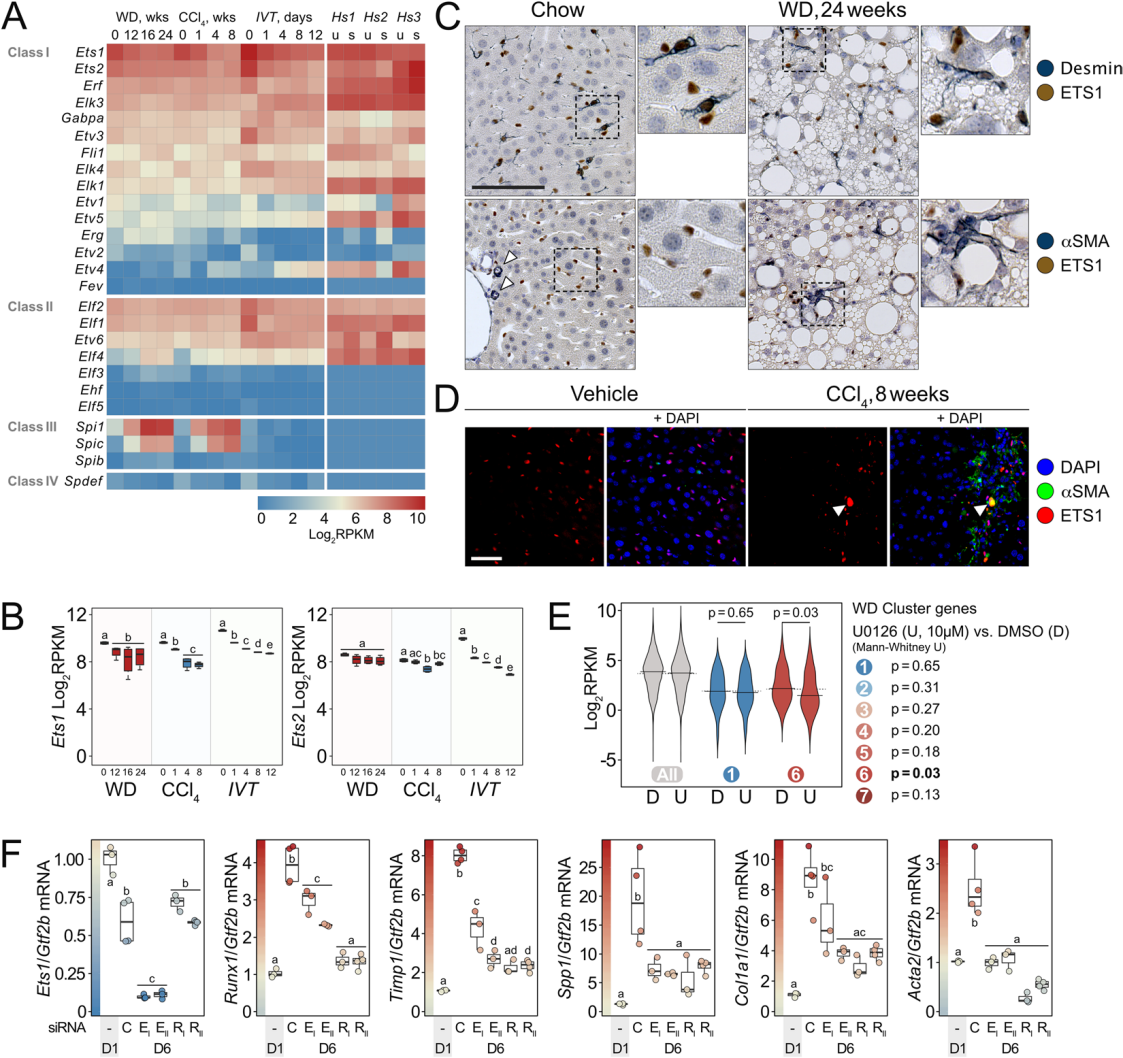
Loss of ETS1 and RUNX1 functions abates HSC transcriptional plasticity. (**A**) Heat map of unscaled log_2_RPKM values for 26 murine ETS TF genes across all three experiments and average log_2_RPKM values of human paralogues in cultured, stimulated (s) or unstimulated (u) human HSCs (*Hs*). *HS1*: GSE68108; *HS2*: GSE78853; *HS3*: GSE101343. Within each class, genes are ordered by *in vivo* expression averages. (**B**) Log_2_RPKM of *Ets1* and *Ets2* genes through all three experiments. Means within experiments not sharing a letter are significantly different (FDR <0.05, edgeR exact test for overdispersed data). (**C**) Double IHC of ETS1 (brown) and Desmin (blue, top row) or αSMA (blue, bottom row) on FFPE liver sections from mice fed WD for 24 weeks or chow (scale bar 100 μm). Small images show 2.5x magnifications of indicated areas. White arrows indicate αSMA^+^ media myocytes as staining control. (**D**) IF of ETS1 and αSMA in liver sections from CCl_4_- (8w) and vehicle-treated mice with or without DAPI. Arrows indicate non-specific signal from an autofluorescent cell (scale bar 50 µm). Background fluorescence was reduced using Huygens Software v. 16.10. (**E**) Log_2_RPKM values of genes (avr. RPKM >1) in mouse HSCs cultured for 6 days in the presence of DMSO (D) or 10 μM U0126 (U). Bean plots shown for all genes, genes belonging to WD cluster 1 or 6, respectively and p-values (U0126 vs. DMSO) shown for all WD clusters. (**F**) qPCR analysis of primary, siRNA-transfected murine HSCs (C: Universal control siRNA, E_I_/E_II_: siEts1 I and II, R_I_/R_II_: siRunx1 I and II). Transfected cells harvested on day 6 post isolation (D6) and mRNA levels of indicated genes normalized to *Gtf2b* expression and day-1 controls (D1). Means not sharing a letter are significantly different (p_adj_ <0.05, ANOVA, Tukey-adjusted). All values are averages of 3–4 biological replicates.

The 26 murine ETS family members are grouped into four classes by their *in vitro* DNA binding specificities^43^ but exhibit redundant binding determined by promoter context^44–46^. To narrow down candidate factors that could induce genes through putative class-I ETS TF binding sites in HSCs we assessed expression levels of all Ets family members in our models. *Ets1* and *Ets2* have the highest overall expression levels (Fig. 4A) with both being reduced upon HSC activation (Fig. 4B). Class III family members *Spi1* (*PU.1*) and *SpiC* appear induced upon HSC activation *in vivo* but are absent *in vitro*. Both TFs are known drivers of myeloid cell specification^47^ and are highly expressed in macrophages. They may either be selectively induced in HSCs *in vivo* or reflect traces of macrophage mRNA in our HSC preparations. The latter and more likely possibility again underscores the importance of applying the *IVT* model as ‘filter’ in our analyses. Comparisons of ETS family expression profiles to available datasets from cultured and passaged human HSCs reveal strict conservation across species (Fig. 4A; *Hs1-3*). hETS1 and 2 are highly expressed together with the class-I members hELK1, hELK3, hERF, and hETV5 whereas the class-III and -IV family members are not expressed in cultured human HSCs.

Double-IHC analyses of livers from WD- and chow-fed mice clearly demonstrate co-expression of nuclear ETS1 and cytosolic Desmin (Fig. 4C top). Desmin is an established marker of rodent HSCs^48,49^ here enclosing the characteristic cytosolic lipid droplets. Importantly, in livers from WD-fed mice, nuclear ETS1 is found in αSMA^+^ cells (Fig. 4C bottom). ETS1^+^/αSMA^+^ cells appear closely associated with the crown-like structures and themselves form structures around highly lipophagic hepatocytes. ETS1^+^ cells expressing neither Desmin nor αSMA are seen in all sections. Based on their tissue distribution we deduce that these are endothelial cells.

We readily detect nuclear ETS1 protein also in livers from CCl_4_-treated mice (Fig. 4D). Nuclear ETS1 expression is restricted to non-parenchymal cells mainly in areas of αSMA expression. Taken together, the consistent detection of nuclear ETS1 in both quiescent and activated HSCs in livers of our two *in vivo* models supports the involvement of ETS1 in NASH-associated HSC activation.

We finally wanted to test our premise by blocking ETS1 and RUNX1 functions and assess if HSC plasticity was compromised. The reported redundancy between ETS family members led us to initially intervene in MEK/ERK signaling immediately upstream of ETS1^50^. To examine the direct effects on HSCs, we exposed isolated HSCs to the selective MEK inhibitor U0126^51^ for six days during *in vitro* activation and surveyed global gene expression. Focusing on genes expressed *in vitro* (avr. RPKM >1), we evaluated which of the identified WD clusters 1–7 were impacted by MEK inhibition. In keeping with our hypothesis, MEK inhibition significantly repressed the induction of WD cluster 6 (p < 0.03; Fig. 4E) but had no significant effects on the other clusters. These are independent tests of individual clusters and p-values are hence not adjusted for multiple testing. The effects of MEK inhibition on HSC activation-markers, proliferation-associated genes, and key ETS1 motif-containing genes are shown along with gene cluster affiliation and ETS1 motif position in Supplementary Fig. 4C. The expression of HSC activation markers *Timp1*, *Spp1*, *Mmp3*, and *Gas6* was significantly reduced upon MEK inhibition (FDR <0.05) whereas the reduction in *Acta2* and *Col1a1* expression failed to reach significance. Unlike *Col1a1*, expression of other collagens exemplified by *Col3a1* and *Col5a2* showed no tendency toward repression. As expected, expression of *Egr1*, *Ccnd1*, *Top2a*, *Cenpe*, and *Rrm2* was repressed by U0126 indicating a steep decline in proliferation capacity.

We then proceeded with siRNA-mediated knockdown of ETS1 and RUNX1. Primary HSCs were transfected with siRNAs two days post isolation and harvested 96 hours later. Two siRNA oligos were used for each TF. RT-qPCR quantification of mRNA shows that selective knockdown of either ETS1 or RUNX1 potently blocks the induction of myofibroblast genes (Fig. 4F, p_adj_ <0.05) including *Timp1* and *Spp1* of cluster 6. Notably, ETS1 knockdown also impedes the induction of the *Runx1* gene (p_adj_ <0.05). These acute effects agree with the effects of MEK inhibition and demonstrate the direct involvement of ETS1 and RUNX1 in HSC activation. The very low expression of *Spi1* (PU.1) in HSCs was unaffected by knockdown of either ETS1 or RUNX1 suggesting that HSC PU.1 is not involved in the activation process *in vitro* (Supplementary Fig. 4D). Interestingly, *Col3a1* and *Col5a2* expression was reduced upon ETS1 and RUNX1 knockdown, despite being unaffected by U0126. This could be explained by differences in the timing and efficiency of ETS1 repression as well as their specific promoter and enhancer compositions. Both *Col3a1* and *Col5a2* have promoter-proximal ETS1 motifs.

We have demonstrated that ETS1 and RUNX1 motifs are strongly enriched in promoters of the induced genes in cluster 6 and highly predictive of NASH-associated gene induction in HSCs. This together with the nuclear localization of ETS1 in activated HSCs of NASH livers and the attenuation of HSC activation by ETS1 and RUNX1 knockdown or MEK inhibition denotes a conserved MEK-ERK-ETS1-RUNX1 signaling axis promoting HSC transcriptional plasticity and activation in NASH.

## Discussion

We here show that Western diet and fructose feeding of C57BL/6J mice is a pathophysiologically relevant NASH model recapitulating characteristics of human disease while inducing profound transcriptional changes in HSCs. Specifically, we identify ETS1 and RUNX1 as probable transcriptional drivers of HSC activation in murine NASH. Despite etiological and histopathological differences between WD-fed and CCl_4_-treated mice, we find transcriptional programs underlying HSC activation and fibrogenesis being remarkably similar across disease models. As such, our results point to HSCs as source of hepatic myofibroblasts in NASH and to HSC activation as a point of convergence in the development of chronic liver disease of diverse etiologies.

ETS1 is a known transducer of growth factor and ERK signaling controlling genes involved in inflammation, proliferation, and ECM remodeling^24,52–55^ and has previously been detected in HSCs^55,56^. ETS1 is permissive of TGFβ signaling to Smad2/3-regulated genes in HaCaT keratinocytes, where 56% of Smad2/3-binding regions also contain ETS1 motifs^57^. A recent study further identifies ETS1 as a transcriptional amplifier releasing paused RNA polymerase II genome-wide in response to VEGF signaling in human umbilical vein endothelial cells^58^. As such, a central role for ETS1 in NASH-associated HSC plasticity would be consistent with the established importance of TGFβ signaling in HSC activation. By contrast, the ETS family member ERG counteracts endothelial TGFβ-SMAD3 signaling and protects against endothelial-to-mesenchymal transition and fibrosis in the mouse liver^59^. These reports demonstrate the intrinsic differences between ETS family members and highlight the cell type-specificity by which the ETS family transmits growth factor signals to the genome.

Cell type-specific responses to growth factors may hence reflect relative levels of ETS family members but also the activities of cooperating TFs. Other signal-dependent TFs could cell type-specifically interact with *transcriptional amplifiers* of the ETS family to induce context-specific gene programs. Indeed, whereas the broad induction of genes with ETS1 motifs in their promoters across models of HSC activation is consistent with ETS1 serving as transcriptional amplifier, the presence of both ETS1 and RUNX1 motifs is the better predictor of gene induction during HSC activation. In HSCs, part of the transcriptional response to growth factors may therefore be defined by ETS1 and RUNX1 co-occupancy at promoters controlling signature processes of HSC activation including *cell adhesion*, *cell motility* and *proliferation*. ETS1 has previously been shown to act cooperatively with RUNX1 through complex formation and mutual derepression^46,60,61^ and some studies even identified composite ETS1-RUNX1 binding sites. While the average spacing of ETS1 and RUNX1 motifs in our study of 77 bps would accommodate ETS1-RUNX1 interactions we do not find composite binding motifs in the analyzed promoters. Rather, the enriched ETS1 and ETS1-*like* motifs are canonical *in vivo* binding motifs of class-I ETS TFs^43^ and a role for RUNX1 in stabilizing higher-order TF complexes at proximal ETS1 binding sites can be envisioned. This would be in keeping with previous reports on RUNX1 and ETS TFs in hematopoiesis and T-cell specification^62–65^. Whether the reduction in *Ets1* expression after HSC activation seen here and elsewhere^56^ affects already activated genes, results in transient gene induction, or is required for full target gene activation as suggested in other settings^66^ is yet to be explored in the context of NASH.

While our current study finds strong correlation between promoter TF motifs and HSC gene induction in NASH, we presume that extensive reconfiguration of enhancer-promoter interactions contributes to the observed HSC transformation. The nature of these interactions should be explored further as should genome-wide interactions with other TFs including bZIP-TFs. Although promoter AP-1 motifs are overall less predictive of strong HSC gene induction than ETS1 and RUNX1 motifs, AP-1 may convey growth factor signals to NASH- and proliferation-associated genes in and beyond cluster 6 and together with ETS1 account for the full effects of MEK/ERK inhibition. ETS1 and RUNX1 do regulate a defined subset of genes induced upon HSC activation across models, but our data suggests that they also engage in gene regulatory networks beyond cluster 6. Such networks likely bring together both general and disease-specific regulators and help coordinate the transcriptional rewiring of HSCs over time. Functional interactions between transcriptional regulators and transcriptional cofactors in NASH will therefore be investigated in primary HSCs by chromatin immunoprecipitation-sequencing assays currently under development.

Our study demonstrates the advantage of cell type-resolved analyses by providing novel insights into HSC activation *in vivo*, which were missed even in comprehensive transcriptomic studies of whole tissue^33,35^. Routine cell type-resolved transcriptomic analysis of patient biopsies is currently not feasible, but reference gene expression signatures of individual hepatic cell populations during NASH progression would help deconvolve the composite expression profiles of complex patient specimens. This should give *richer* datasets more accurately reflecting disease stage and offering higher diagnostic and prognostic power. We utilized cellular retinoid content for HSC purification by density gradient centrifugation and flow cytometry^67^. This has the obvious limitation that retinoid-depleted HSCs and myofibroblasts were not recovered for gene expression profiling. Our current study hence offers novel insights into the plasticity of HSCs during NASH development but likely underestimates the shift in HSC gene expression in advanced NASH. Application of distinct models of HSC activation helped us separate common transcriptional events shaping the coordinated response to fibrogenic signals from experimental noise and artifacts. Nonetheless, actual differences in gene expression do exist between our models reflecting the differences in biology and experimental conditions. In particular, differences between *in vitro* and *in vivo* activation are captured in PC1 of our principal component analysis (Supplementary Fig. 2C) and align with previous studies of human^32^ or mouse^29^ HSCs activated *in vivo* and *in vitro*. As is the case in our study, differences are of both quantitative and qualitative nature.

In conclusion, our findings implicate HSC activation in the pathophysiology of a relevant animal model of human NASH and strongly support roles for ETS1 and RUNX1 TFs as drivers of HSC activation and plasticity. The conserved transcriptional response in HSCs to hepatic insult emerges as a point of convergence of upstream signaling events across etiologies. Cell type-resolved studies like ours may hence inform clinical interrogation of the transcriptional landscape promoting human myofibroblast formation in NASH and beyond and help refine diagnostics and risk stratification.

## Methods

### Animal experiments

Male C57BL/6JBomTac mice were housed under standard conditions, under a 12:12-hr light/dark cycle, with unrestricted access to food and drinking water (exceptions are outlined below). All animal experimentation was approved by the Danish Animal Experiment Inspectorate (approval #2015-15-0201-00550) and complied with the ARRIVE guidelines. To induce NASH/hepatic fibrosis mice were either fed with Western diet (WD) for up to 24 weeks or gavage treated with CCl_4_ up to 8 weeks. *WD experiments*: mice were fed WD (829100, SDS Research Diet) supplemented with fructose (42 g/L) in drinking water for 6, 12, 16, or 24 weeks. Control mice received standard chow diet (altromin #1324) and regular drinking water. WD feeding was initiated so that all mice were 30 weeks of age at the end of the experiment. *CCl*_4_ *treatment*: After over-night (o.n.) fasting, mice were given CCl_4_ (1 mL/kg; 289116, Sigma-Aldrich) diluted in corn oil at a 1:4 ratio twice weekly by oral gavage for 1, 4, or 8 weeks. Control mice received same amount of corn oil as the CCl_4_-treated mice and were fasted accordingly. Every second week, WD-fed mice were fasted o.n. (fructose water was substituted with normal drinking water), weighted, and fasting blood glucose was measured using the FreeStyle Freedom Lite apparatus (Abbott Diabetes Care Inc.). For gene expression analysis, three to four mice were used per condition. For immunohistochemistry and histopathology, two mice were used per condition.

### Hepatic Stellate Cell isolation

Primary hepatic stellate cells (HSCs) were isolated from male C57BL/6JBomTac mice essentially as described in^67^. In brief, mice were opened surgically under anaesthesia and livers were perfused through the inferior vena cava with Hanks Buffered Salt Solution (HBSS; 14175103, GIBCO) and 0.5 mM EGTA after the portal vein was cut and the upper IVC clamped. After 2 min, livers were perfused with a HBSS/Pronase (10165921001, Roche) solution for 8 min and then HBSS/Collagenase-B (11088831001, Roche) solution for 12 min. The digested livers were removed and minced in HBSS with pronase/collagenase/DNAse-I (10104159001, Roche) and passed through a 70-μm cell strainer. Liver cells were centrifuged and washed twice in Gey’s Balanced Salt Solution (GBSS) followed by density gradient-separation of HSCs using a GBSS/Histodenz (D2158, Sigma-Aldrich) solution. After centrifugation, HSCs located in the interface were collected and washed once in GBSS.

### Flow cytometry

After isolation, live HSCs were fluorescence-activated cell sorting (FACS) sorted for RNA sequencing and *in vitro* cell culture. HSCs were selected based on retinoid autofluorescence using the 407 nm laser for excitation and the 450/40 bandpass filter for detection (FACSAria III, BD Biosciences). Live cells were selected based on live/dead-stain with SYTOX™ Red Dead cell Stain (S34859, Thermo Scientific), which was detected with the 633 nm laser for excitation and the 660/20 bandpass filter.

### *In vitro* HSC culture

FACS-sorted HSCs were cultivated for 2 hrs (control), 12 hrs, 1, 4, 8, or 12 days in technical triplicates in Falcon 12-well Polystyrene culture plates (50000 cells/well) or 8-well uncoated chamber slides (20000 cells/well). HSCs were kept in Dulbecco’s Modified Eagle’s Medium (DMEM; #52-100-021, GIBCO) supplemented with 10% calf serum (B15-005, PAA), 100 U Penicillin, 100 µg/mL Streptomycin (#DE 17-602E, Lonza), and 1.25 µg/mL Amphotericin B (15290-018, Gibco). Control cells were kept in suspension in culture medium for 2 hrs before centrifugation and snap freezing. For MEK inhibition, U0126 (10 µM; #9903, Cell Signaling Technology) or DMSO was added to the medium from day 2 until harvest on day 6 post isolation. Fresh medium was added every two days.

### Transfection with siRNA

HSCs were purified from male wild type C57BL/6JBomTac mice and seeded in Falcon 12-well polystyrene culture plates with full medium (as above). 24 hours post isolation, the first HSCs were harvested for RNA extraction as described below. Medium on the remaining HSCs was changed to DMEM supplemented with 10% calf serum without antibiotics. 48 hours post isolation, HSCs were transfected with 2.5 pmol per 50000 HSCs of the individual siRNAs directed against ETS1 (SASI_Mm01_00158628; SASI_Mm01_00158629) or RUNX1 (SASI_Mm01_00306068; SASI_Mm01_00306069) (Sigma-Aldrich) using Dharmafect-1 (Dharmacon). Control cells were transfected with negative control siRNA oligos (SIC001, Sigma). After 12 hours, full medium with antibiotics was reintroduced and cells were kept in culture until day 6 post isolation before harvest and RNA extraction. Each transfection was carried out with 3–4 biological replicates.

### Immunofluorescence and confocal microscopy

Livers from CCl_4_-treated mice were fixed in PBS, 4% paraformaldehyde (PFA) (P6148, Sigma-Aldrich) for 4 hours, incubated o.n. in PBS/20% sucrose, and embedded in O.C.T. compound (SAKU4583, Sakura). *In vitro*-cultured cells were fixed on ice in chamber slides in PBS, 4% PFA for 10 min. Excess PFA was quenched by adding 50 mM NH_4_Cl for 5 min. Cryo sections (12 μm thickness) and fixed cells were permeabilized using 0.1% Triton-X-100 and blocked in PBS, 1% BSA (81066 N, Millipore), 1% normal Donkey serum (017-000-121, Jackson ImmunoResearch). For protein detection, sections and cells were incubated with FITC-conjugated anti-α-Smooth muscle actin (F3777, 1:500, Sigma-Aldrich) and DAPI (0.5 mg/ml, D9542, Sigma-Aldrich). Additionally, Cryo sections were probed with rabbit-anti-Ets1 (D8O8A, 1:1000, Cell Signaling Technology) or rabbit-anti-Desmin (RB-9014-P0, 1:100, Thermo Scientific) followed by secondary AlexaFluor647-labeled donkey-anti-rabbit IgG (711-605-152, 1:500, Jackson ImmunoResearch). Confocal microscopy was done on the Zeiss LSM510 Meta instrument using the LSM 4.2 acquisition software and image deconvolution was performed in Huygens Software v. 16.10.

### Immunohistochemistry (IHC), Hematoxylin-Eosin (HE) staining and Picrosirius Red staining

Livers from CCl_4_-treated and WD-fed mice were fixed in PBS, 4% PFA for 20 hours and embedded in paraffin. FFPE blocks were cut in 6 μm sections and deparaffinized before IHC, HE (HT110232 and MHS16, Sigma-Aldrich), or Picrosirius red staining (Department of Pathology, OUH, Denmark). For IHC, sections were incubated with Dual endogenous enzyme block (S2003, DAKO) followed by incubation in Citrate buffer, pH 6.0 (C9999, Sigma-Aldrich) at 60 °C. Sections were probed with rabbit-anti-α-SMA (ab150301, 1:200, Abcam) or rat-anti-F4/80 (Ab6640, 1:500, Abcam) followed by rabbit-anti-rat IgG (ab102248, 1:200, Abcam). For detection, all sections were incubated with EnVision-HRP coupled to goat-anti-rabbit IgG (K4003, Dako) and incubated with AEC + Substrate-Chromogen solution (K3461, Dako). Double immunostaining was performed as previously described^68^. After rehydration, sections were boiled in Tris-EGTA (pH 9.0) and incubated in PBS with 0.6% H_2_O_2_/50 mM NH_4_Cl. Sections were probed with rabbit anti-ETS1 (D8O8A mAb, #14069, 1:200, CST) followed by HRP-conjugated secondary antibody (P448, Dako). The DAB chromogen (Sigma, D5637) was used for detection. Thereafter, sections were re-boiled in Tris-EGTA and probed with rabbit anti-Desmin (ab15200, 1:200, Abcam) or rabbit anti-α-SMA (ab150301, 1:200, Abcam) followed by ImmPRESS Polymer HRP Reagent (MP-7451, Vector Lab.) and detection by VECTOR SG chromogen substrate (SK-4700, Vector Lab.). After antibody staining, a hematoxylin counterstain was performed. Slides were scanned using The NanoZoomer-2.0HT digital slide scanner (Hamamatsu).

We staged liver fibrosis according to the model developed for human NAFLD by Kleiner *et al*.: F0 is no fibrosis, F1A is mild perisinusoidal fibrosis in zone 3 only, F1B is moderate perisinusoidal fibrosis in zone 3 only, F1C is portal or periportal fibrosis only, F2 is perisinusoidal fibrosis in combination with portal or periportal fibrosis, F3 is bridging fibrosis and F4 is cirrhosis^4^. We also assessed liver histology according to the nonalcoholic fatty liver disease activity score (NAS-CRN) for steatosis (0–3). Lobular inflammation was assessed as described by Liang *et al*.^69^ for NAFLD mouse models, by counting the number of inflammatory foci per field of view (3.1 mm^2^).

### Coherent anti-strokes Raman scattering spectroscopy (CARS) and second harmonic generation (SHG) microscopy

Livers from WD-fed mice were excised and immediately frozen in OCT compound. Frozen sections were cut at 20 μm thickness and mounted with PBS. For detection of lipids, a Raman frequency at 2855 cm^–1^ for CH_2_ symmetric stretch vibration was applied (Leica TCS SP8 microscope). The wavelengths of the pump and stokes beams were 816.4 nm and 1064.6 nm, respectively. For SHG the excitation, wavelength was set to 816.4 nm in the investigation of fibrilar collagen.

### Isolation of RNA, qPCR, and RNA sequencing

Purified HSCs were sorted into RLT lysis buffer from RNeasy Mini Kit (74106, Qiagen) and total RNA extracted according to manufacturer’s instructions after standard phenol/chloroform extraction. *In vitro* cultured HSCs were harvested in Isol RNA Lysis Reagent (2302700, 5PRIME) and RNA extracted as above. Whole liver RNA from male C57BL/6JBomTac mice was extracted from pulverized snap-frozen livers. RNA concentrations were measured using a Qubit 3.0 Fluorometer (Invitrogen) and quality of the RNA was assessed on a Bioanalyzer 2100 (Agilent Technologies). For qPCR, 20 ng purified RNA was reverse transcribed using the iScript Select cDNA synthesis-kit (1708897, Biorad) and amplified using FastStart Essential DNA Green Master mix (6924204001, Roche) on a Lightcycler 480 II instrument (Roche). Primer sequences are shown in suppl. table T4. For RNA sequencing, purified RNA (3–4 biological replicates) was polyA-selected and subjected to fragmentation before cDNA synthesis. For library construction, we used the NEBNext Ultra RNA Library Prep Kit for Illumina and sequenced using the Illumina HiSeq. 1500 platform.

### RNA-seq data analysis

Star^70^ was used to align reads to the mm10 *Mus musculus* genome. EdgeR^71^ was used through the Homer^42^ pipeline for analysis of differential gene expression using default settings. The Cytoscape app. TiCoNE^41^ was used for time-course clustering of the 870 *common* genes by average RPKM values subjected to variance-stabilizing transformation and scaling. Expression profiles were clustered by Pearson’s correlation coefficients using PAMK clustering. The 10% least fitting genes and the smallest cluster from each experiment (6 and 15 genes, respectively) were removed *post-hoc*. We used Homer^42^ and default settings herein for motif-finding including motif-scoring by hypergeometric distribution and all confidently annotated mouse genes as background set. Weblogo^72^ was used for the generation of motif logos. Metacore (Clarivate Analytics) was used for gene ontology analyses using all expressed genes as background list. Data visualization and statistical analyses were performed in R. For differential gene expression, FDR <0.05 is regarded statistically significant unless stated otherwise. Gene expression datasets of human HSCs were retrieved from the GEO repository GSE68108^26^, GSE78853^27^, and GSE101343^73^ and mapped to the hg38 *Homo sapiens* genome.

### Data files

Raw and processed sequencing data files have been uploaded to the Gene Expression Omnibus (accession no. GSE116987) and EBI ArrayExpress repository (accession no. E-MTAB-7054).

### Statistical analysis

When comparing the weight and fasting blood glucose for WD- and control-fed mice, Tukey-adjusted one-way ANOVA was used to determine significance. p_adj_ <0.05 was regarded as significant difference. Weight data are shown as mean ± standard deviations (SD). For gene expression data, significant differences were calculated using one-way ANOVA (Tukey-adjusted for multiple testing) or the Mann-Whitney U-test/Wilcoxon Rank-sum test (Benjamini-Hochberg adjustment) as indicated.

## Supplementary information

Supplementary figures and tables
