# Supplementary material for: Transcriptional regulation of Hepatic Stellate Cell activation in NASH
Source: Sci Rep. 2019 Feb 20;9:2324. doi: 10.1038/s41598-019-39112-6 (PMC6382845; doi:10.1038/s41598-019-39112-6)
Supplement: Supplementary file 1 — Supplementary figures and tables [file 41598_2019_39112_MOESM1_ESM.pdf]

Transcriptional regulation of Hepatic Stellate Cell activation in NASH

Ann-Britt Marcher, Sofie M. Bendixen, Mike K. Terkelsen, Sonja S. Hohmann, Maria H. Hansen, Bjørk D. Larsen, Susanne Mandrup, Henrik Dimke, Sönke Detlefsen, and Kim Ravnskjaer

Supplementary information:

Supplementary figure 1

Supplementary figure 2

Supplementary figure 3

Supplementary figure 4

Supplementary table T1

Supplementary table T2

Supplementary table T3

Supplementary table T4

Supplementary table T5

LEGENDS

Supplementary Fig. 1

a IF confocal microscopy of liver sections from ctrl and CCl_4-treated mice (0 and 8 weeks) stained for Desmin and αSMA (scale bar 50µm). Background fluorescence was reduced using Huygens Software v. 16.10.

b IF confocal microscopy of isolated mouse HSCs transdifferentiating *in vitro* 1, 4, and 12 days post isolation and stained for αSMA (scale bar 50µm). Background fluorescence was reduced using Huygens Software v. 16.10.

c Phase contrast microscopy of isolated mouse HSCs transdifferentiating *in vitro* 12 hours to 12 days post isolation (scale bar 50µm). Insert at 12 hours image showing 10x magnification of a single cell.

d Representative FACS plot showing sequential gating for vitamin A^+ cells, singlets, and live HSCs enriched from mouse liver cell suspension by density gradient centrifugation. Number of positive cells is indicated as fraction of parent population. Purity of the sorted cells is verified by microscopic evaluation.

e Coherent anti-Stokes Raman scattering (CARS) spectroscopy and second harmonic generation (SHG) microscopy of liver sections from Western diet-fed mice (scale bar 50µm).

f Hematoxylin and Eosin (H&E), sirius red staining, F4/80 IHC, and aSMA IHC of FFPE liver sections from CCl_4-treated mice (scale bars 100µm).

Supplementary Fig. 2

a Cumulative distribution of RPKM values for all genes in three experimental models of HSC activation; WD feeding (24w vs. chow), CCl_4 treatment (8w vs. ctrl), and IVT (12d vs. ctrl). Kolmogorov–Smirnov tests were used to calculate corresponding D and p-values for pairwise comparisons of distributions. D is the maximal difference between distributions. Expression values represent averages of 3-4 biological replicates.

b Heat map of z-scores from 3913 genes induced or repressed ≥4-fold (FDR < 0.05) in any experimental model and with median rlog expression across all samples of ≥5. Samples and genes subjected to unsupervised hierarchical clustering. Scaling performed across all replicates in heatmap.

c PCA plot based on all regulated genes in any experiment ($log_2FC \neq 0$; FDR < 0.01, $RPKM_{avr} > 20$). All replicates shown as small spheres. Bigger spheres indicate centroids of replicates.

d Heat map of 2484 genes, which are induced ≥2-fold (FDR<0.05) between any two time points in all three models, or repressed ≥2-fold (FDR<0.05) between any two time points in all three models. Exon counts are individually scaled for each model. Expression values represent averages of 3-4 biological replicates.

e Log_2RPKM of HSC activation markers through all three experiments. Means within experiments not sharing a letter are significantly different (FDR < 0.05, edgeR exact test for overdispersed data).

Supplementary Fig. 3

a Significance of motif enrichment for all genes changing expression (-2 ≥ log2FC ≥ 2; FDR < 0.05) between any two time points in our models. Motif enrichment analyses were performed on genes with reduced (red) and increased expression (green), all genes (dark) and model-*specific* genes (light). Motifs are grouped according to Pearson's correlation.

b Comparison of RPKM values for select hepatocyte TFs and HSC marker genes in isolated HSCs from 24-week WD- or chow-fed mice and whole liver (Liv.). RPKM values are normalized to whole liver expression (n=3). Means not sharing a letter are significantly different (FDR < 0.05, edgeR exact test for overdispersed data).

c Motif correlation matrix showing known motifs from Homer motif enrichment analyses subjected to hierarchical clustering by their Peason's correlation.

Supplementary Fig. 4

a Log_2FC of the 870 *common* genes in WD and CCl_4 experiments grouped by the presence of putative Ets1 and Ap1 binding sites or both (E+A). Dashed horizontal lines indicate average log_2FC for all *common* genes in experiment. Means not sharing a letter are significantly different (FDR<0.05, pairwise Wilcoxon rank-sum test, Benjamini-Hochberg corrected).

b Log_2 fold enrichment of Ets1 (E), Runx1 (R), and dual motifs (ER) in promoters of genes falling into GO-SLIM categories and respective WD clusters where each GO-SLIM category is enriched. Enrichment calculated relative to all regulated genes in GO-SLIM analysis.

c Comparison of RPKM values for key ETS1/ETS1-*like* motif-containing genes, HSC activation markers, and proliferation-associated genes in mouse HSCs cultured for six days in the presence of 10 μM U0126 (blue) or DMSO (red) (n=3). WD cluster # and position of motifs relative to TSS indicated for each gene. * FDR < 0.05, edgeR exact test for overdispersed data. RPKM values normalized to DMSO controls.

d qPCR analysis of primary, siRNA-transfected murine HSCs (C: Universal control siRNA, E_I/E_{II}: siEts1 I and II, R_I/R_{II}: siRunx1 I and II). Transfected cells harvested on day 6 post isolation (D6) and mRNA levels of indicated genes normalized to *Gtf2b* expression and day-1 levels (D1). Whole liver cDNA (L) was prepared form healthy, male C57BL6/J mice. Means not sharing a letter are significantly different ($p_{adj} < 0.05$, ANOVA, Tukey-adjusted).

Supplementary Tables

T1 Diet nutrient and energy composition of Western and chow diets. AFE = Atwater Fuel Energy.

T2 Gene cluster information from Western diet (WD) and CCl_4 experiments. Pearson's correlation calculated for gene profiles within clusters and for cluster prototypes across experiments. Hypergeometric probabilities are calculated for cluster overlaps between experiments.

T3 Homer motif logos, binding factors, and motifs enrichments in promoters of overlapping genes of WD and CCl_4 clusters 6. Ets-family TF, bZiP/AP1, and Runx motif names shaded in blue, green and red, respectively.

T4 List of primers used for RT-qPCR.

T5 List of all *common* genes, genes of WD cluster 6 (blue), and *shared* genes between WD and CCl4 clusters 6 (green).

SUPPLEMENTARY FIGURE 1

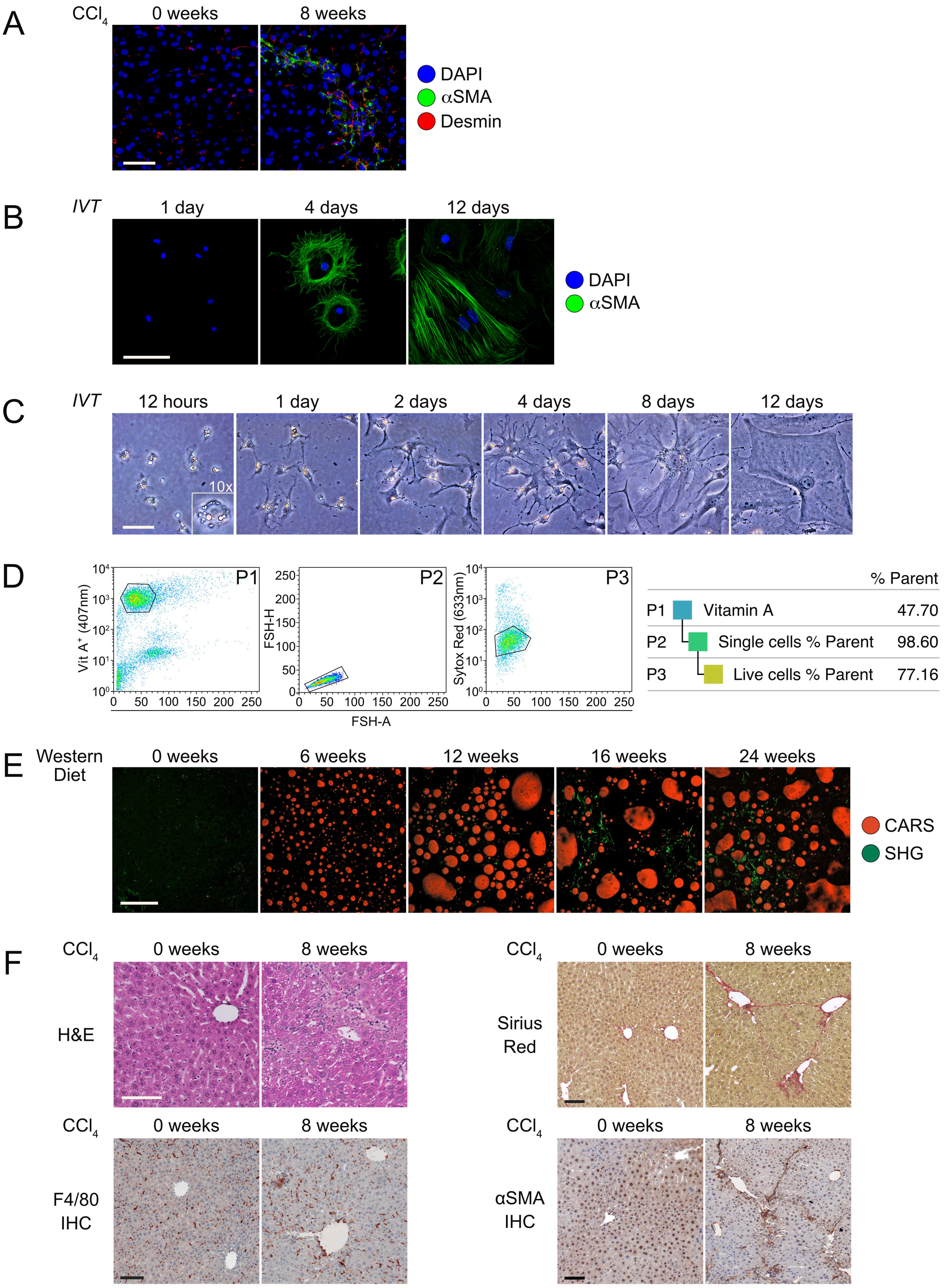

SUPPLEMENTARY FIGURE 2

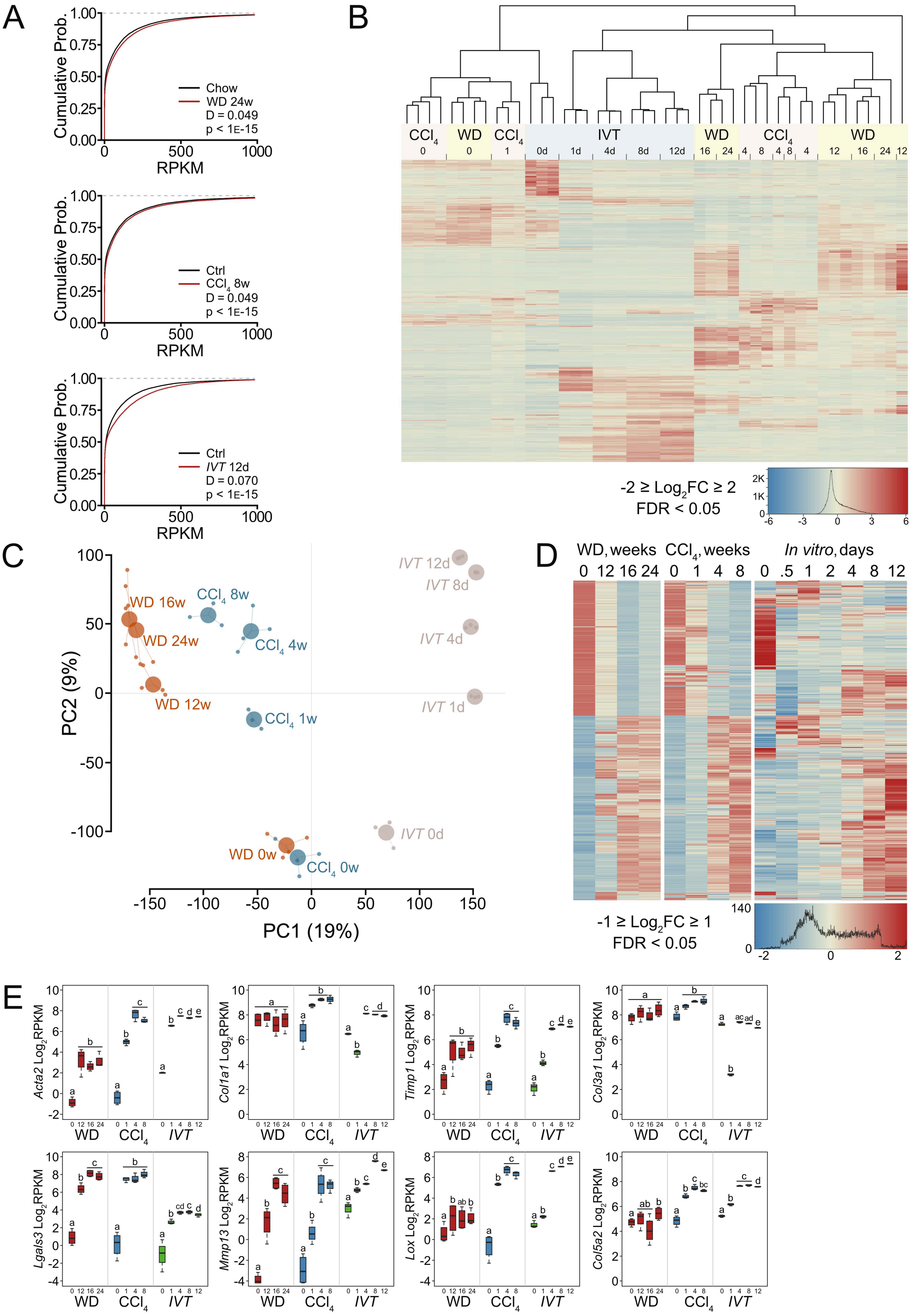

SUPPLEMENTARY FIGURE 3

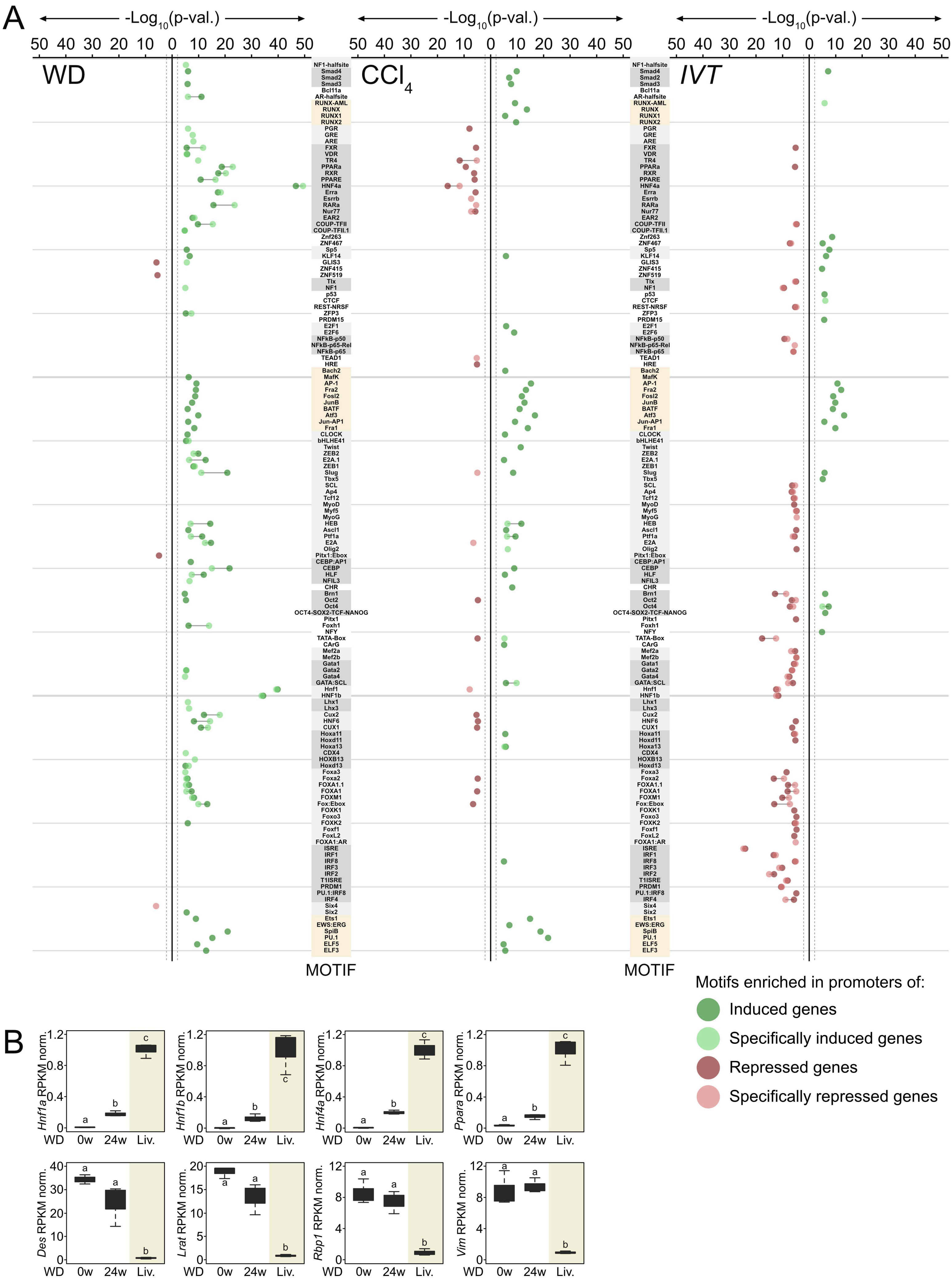

SUPPLEMENTARY FIGURE 3 cont.

C

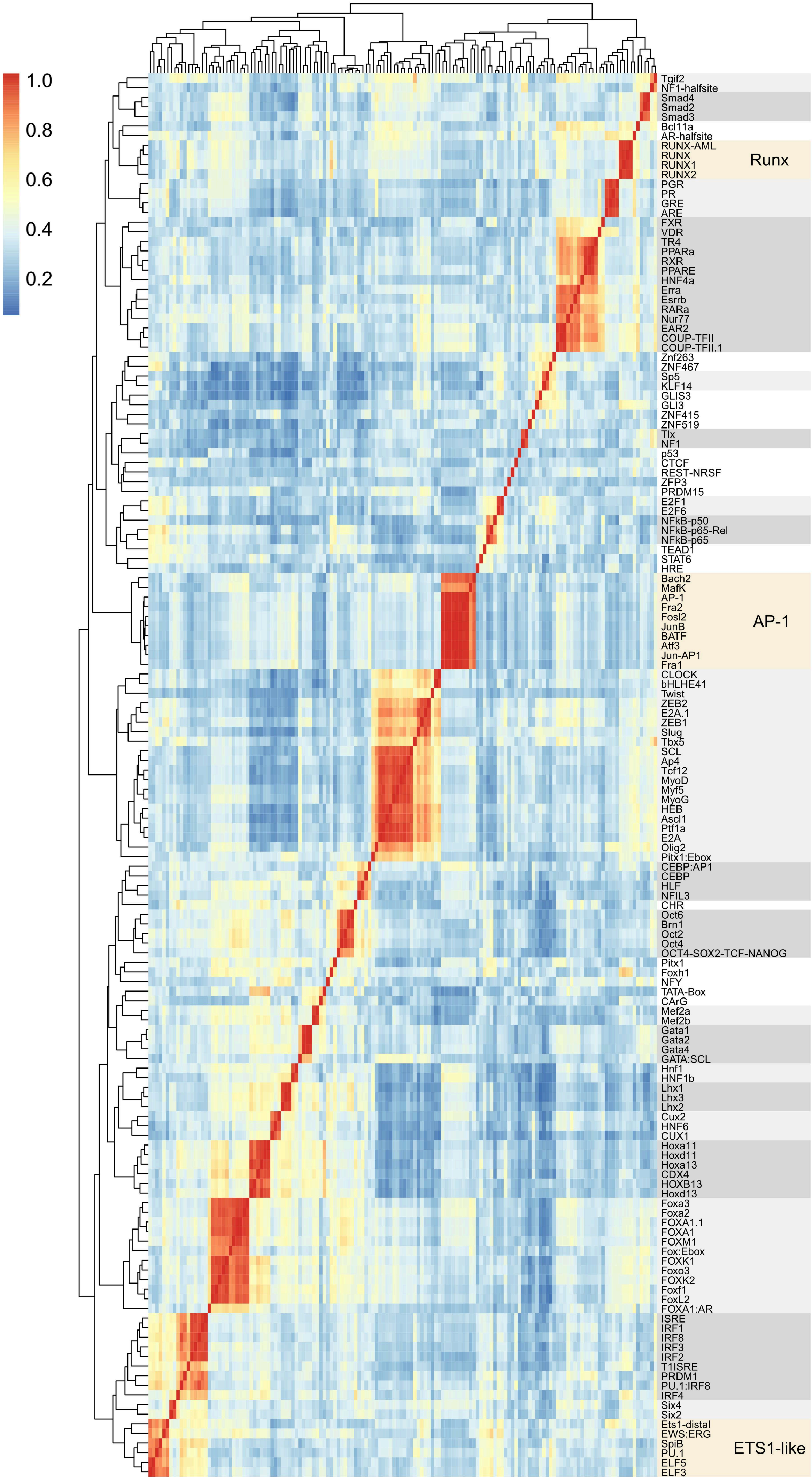

SUPPLEMENTARY FIGURE 4

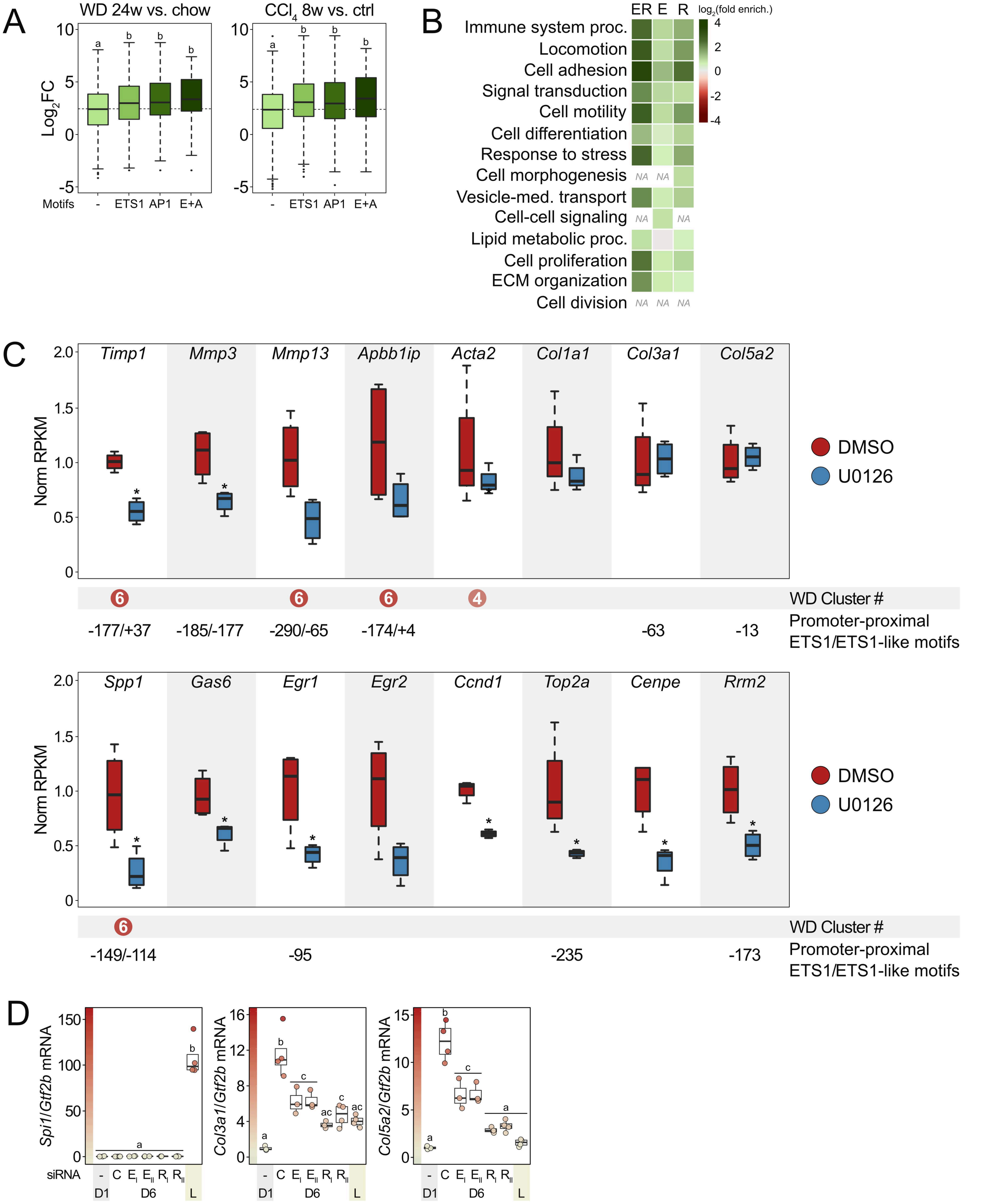

SUPPLEMENTARY TABLES

T1

DIET: **WD**	829100 - 'Western RD'		
SPECIFICATION	%(w/w)	kcal/g	% kcal
Crude Fat	21.4	1.93	42
Crude Protein	17.5	0.70	15
Crude Fibre	3.5		
Ash	4.1		
Carbohydrate	50.0	2.00	43
Total AFE		4.63	100

Cholesterol	0.15		

DIET: **Chow**	Altromin 1324 #30404		
SPECIFICATION	%(w/w)	kcal/g	% kcal
Crude Fat	4.1	0.37	11
Crude Protein	19.2	0.77	24
Crude Fibre	6.1		
Ash	5.9		
Carbohydrate	43.9	2.09	65
Total AFE		2.89	100

T2

WD			CCl_4			Overlap			
Cluster #	Gene count	Pearson corr.	Cluster #	Gene count	Pearson corr.	Pearson corr.	Gene count	% of WD cluster	Hypergeometric probability
**1**	174	0.98	**1**	175	0.97	0.99	156	90	1.59E-119
**2**	50	0.97	**2**	67	0.94	0.97	11	22	1.58E-03
**3**	40	0.95	**3**	62	0.95	1.00	9	23	2.13E-03
**4**	54	0.96	**4**	44	0.94	0.94	5	11	1.07E-01
**5**	153	0.97	**5**	90	0.96	0.90	12	13	3.13E-03
**6**	236	0.98	**6**	217	0.97	0.98	142	65	3.36E-38
**7**	70	0.98	**7**	113	0.96	1.00	25	36	1.94E-06
Total	777		Total	768		Total	360		

T3

Rank	Motif Logo	Name	p-value	log_{10}(p-val.)	q-value	% of genes with motif	
						shared	background
1	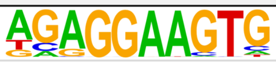	PU.1	1.00E-08	20.3	0	30.30%	10.95%
2	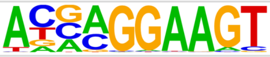	ELF5	1.00E-06	15.5	0	34.85%	16.34%
3	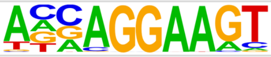	ELF3	1.00E-06	14.1	0.0001	31.82%	14.84%
4	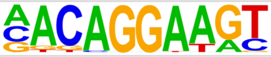	ETS1-distal	1.00E-05	13.2	0.0002	18.94%	6.61%
5	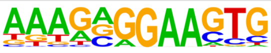	SPIB	1.00E-05	12.6	0.0003	15.91%	5.06%
6	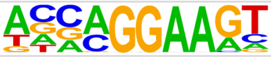	EHF	1.00E-04	1.02	0.0024	43.18%	26.83%
7	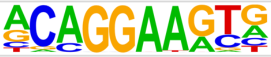	ERG	1.00E-04	9.37	0.0044	50.76%	34.41%
8	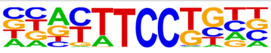	ETV2	1.00E-03	8.52	0.0091	36.36%	22.41%
9	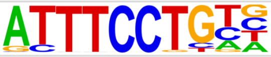	EWS:ERG-fusion	1.00E-03	8.26	0.0105	23.48%	12.24%
10	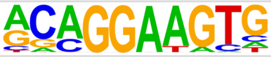	ETS1	1.00E-03	7.98	0.0125	39.39%	25.54%
11	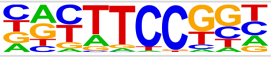	FLI1	1.00E-03	7.43	0.0196	43.18%	29.49%
12	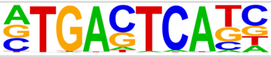	AP-1	1.00E-02	6.79	0.0341	17.42%	8.74%
13	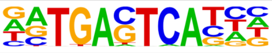	ATF3	1.00E-02	6.20	0.057	15.15%	7.48%
14	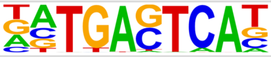	BATF	1.00E-02	6.08	0.0593	15.15%	7.55%
15	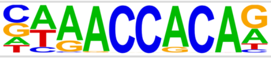	RUNX	1.00E-02	5.95	0.0635	18.94%	10.48%
16	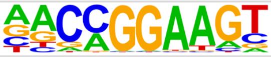	ETV1	1.00E-02	5.58	0.0857	44.70%	33.13%
17	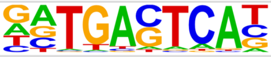	JUNB	1.00E-02	5.07	0.1349	12.88%	6.59%
18	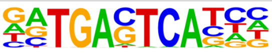	JUN-AP1	1.00E-02	4.85	0.159	7.58%	3.06%
19	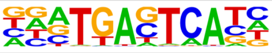	FRA1	1.00E-02	4.69	0.1765	12.12%	6.29%

T4

mGtf2b F	GTTCTGCTCCAACCTTTGCCT
mGtf2b R	TGTGTAGCTGCCATCTGCACTT
mEts1 F	TTGAGATTTTAGGGTTGCGCT
mEts1 R	GGCGGGATGAGAGTAAGTTTG
mRunx1 F	GCACTCTGGTCACCGTCATG
mRunx1 R	GTTGAATCTCGCTACCTGGTTC
mTimp1 F	AACTCGGACCTGGTCATAAGG
mTimp1 R	ACGCTGGTATAAGGTGGTCTC
mSpp1 F	ATCCTTGCTTGGGTTTGCAG
mSpp1 R	CACTGCCAATCTCATGGTCG
mCol1a1 F	CAGGGTATTGCTGGACAACG
mCol1a1 R	GAAGACCAGGGAAGCCTCTT
mActa2 F	CCTGAAGAGCATCCGACACT
mActa2 R	GGTTGGCCTTAGGGTTCAG
mSpi1 F	TCACCCCAAGGGGACTATCT
mSpi1 R	GCCAGCACAAGTTCCTGATTT
mCol3a1 F	TCTGGAAGCCAGAACCATGT
mCol3a1 R	AATGGGATCTCTGGGTTGGG
mCol5a2 F	GTGCTGAAACAGACTGAGGC
mCol5a2 R	CTGAGGTCACTGTAGCACCA

T5

870 *Common* genes
236 WD Cluster 6 genes
142 Shared genes

0610040J01Rik
1600010M07Rik
1700024P16Rik
1700063D05Rik
2310040G24Rik
2810408I11Rik
2810417H13Rik
3110039I08Rik
3300005D01Rik
4632428N05Rik
4930486L24Rik
4930506M07Rik
4931408D14Rik
4933416M07Rik
5033406O09Rik
5430416O09Rik
5430427O19Rik
5430435G22Rik
5730559C18Rik
9130008F23Rik
9130019P16Rik
9930014A18Rik

A630033H20Rik
A830082N09Rik
AA467197
Abca4
Abcb1a
Abcc3
Abcg3
Abi3bp
Ablim3
Ace
Ace2
Ackr1
Ackr2
Ackr4
Acot1
Acot4
Acp5
Acta2
Acvr1c
Acvrl1
Adamts15
Adamts7
Adamtsl4
Adap1
Adcy4
Adcy5
Adcy7
Adgb
Adora2b
Adora3
Adra1a
Adrb1
Adrb2
Adssl1
Afp
Ahrr
AI504432
AI607873
Aim2
Akap12
Akr1c19
Aldh1a3
Aldh1a7
Aldh1b1
Aldh3a1
Aldoc
Alox5
Aloxe3

Als2cr12
Amica1
Amz1
Anapc15
Angpt2
Ank2
Ankrd1
Ankrd33b
Ano1
Ano2
Anpep
Ap1s3
Apbb1ip
Apoa4
Apol9a
Aqp8
Arc
Arhgap11a
Arhgap19
Arhgap22
Arhgap27
Arhgap44
Arhgap9
Arhgef37
Arl11
Armc2
Asb2
Asf1b
Asns
Astn2
Atp13a2
Atp1a3
Atp2a3
Atp2b2
Atp6v0d2
Atp8b4
Avpr1a
B230217O12Rik
B3galnt1
B3gnt3
B3gnt7
B4galnt1
B4galt6
Baiap2l1
Basp1
Batf3
Bdh1
Bdh2

Bdnf
Bend6
Birc5
Bmp4
Bmp6
C1ql1
C230037L18Rik
C5ar1
C5ar2
C77080
C920009B18Rik
Cadm1
Cadm4
Car13
Car9
Caskin1
Casp1
Cav1
Ccdc125
Ccdc136
Ccdc148
Ccdc183
Ccl17
Ccl6
Ccnjl
Ccr1
Ccrn4l
Cd14
Cd200
Cd24a
Cd276
Cd300lb
Cd36
Cd44
Cd53
Cd55
Cd59a
Cd80
Cd83
Cd9
Cd93
Cda
Cdca7l
Cdh1
Cdh4
Cdh5
Cdkn2a
Cdkn3

Cenpp
Cers6
Ces2e
Cgnl1
Ch25h
Chaf1b
Chek2
Chn2
Chst11
Cit
Clec14a
Clec4d
Clec4e
Clhc1
Clmn
Clspn
Cndp2
Cobl
Col23a1
Col25a1
Colec12
Colgalt2
Colq
Comp
Comtd1
Coro1a
Coro2a
Cox6a2
Cox8b
Cpe
Creg2
Crlf1
Crtam
Crybb3
Crym
Csf1r
Cthrc1
Ctla2a
Ctla2b
Ctsh
Cux2
Cx3cr1
Cxadr
Cxcl3
Cxcr4
Cyb561
Cyp7b1
Cystm1

Cyth4
D6Ertd527e
Dbndd1
Dcc
Dchs1
Dclk1
Dctd
Dctpp1
Dennd1c
Dennd2c
Depdc7
Dera
Derl3
Dhh
Dhrs13
Dhrs9
Disp2
Dlgap3
Dll4
Dna2
Dnajb13
Dnase2a
Dnph1
Doc2b
Dock2
Dock5
Dok2
Dsp
Dtl
Dusp13
Dusp14
Dyrk3
Dysf
E2f1
E2f8
Ece2
Edn1
Edn3
Efcc1
Efhd2
Efna1
Egfem1
Egfl6
Egln3
Elmo1
Elovl3
Elovl7
Emb

Emilin2
Enpp6
Entpd1
Epb4.1l3
Epb4.1l4b
Epha4
Erc2
Esam
Esr2
Etv2
Evi2a-evi2b
Evi2b
Exoc3l
F13a1
F630111L10Rik
Fabp4
Fabp5
Fabp7
Fads6
Fam107b
Fam110b
Fam110c
Fam129a
Fam131c
Fam167b
Fam198b
Fam19a1
Fam20c
Fam213a
Fam65b
Fam71a
Fam78a
Fam78b
Fam83a
Fancg
Fat3
Fcgr3
Fcho1
Ffar2
Fgd3
Fgl2
Fhad1
Fhl2
Fkbp11
Fmn1
Fmnl1
Fmo2
Fndc1

Folr1
Foxj1
Foxl1
Frem3
Frmd4b
Frrs1
Fst
Fuca2
Fxyd2
Fyb
Fzd9
Gale
Galnt12
Galnt14
Galnt3
Galnt6
Gas7
Gata5
Gatm
Gcnt1
Gcnt2
Gda
Gdf15
Gdpd5
Gfra2
Ggta1
Gjb3
Gla
Glp2r
Glrp1
Glrx
Glyat
Gm10677
Gm10768
Gm11944
Gm11961
Gm13212
Gm13710
Gm14023
Gm14207
Gm16548
Gm2083
Gm21949
Gm3558
Gm4956
Gm5464
Gm7694
Gm9899

Gmfg
Gmnn
Gna14
Gna15
Gnpda1
Gp49a
Gpnmb
Gpr114
Gpr116
Gpr137b
Gpr137b-ps
Gpr17
Gpr176
Gpr35
Gpr39
Gpr55
Gpr56
Gpr68
Gprasp2
Grhl3
Gria3
Grid1
Grid2ip
Grin2c
Gsg1l
Gsta1
Gsta2
Gsta3
H19
H2-Ab1
H2-DMa
H2-DMb1
H2-Eb1
Havcr2
Hck
Hcls1
Hdac9
Hebp1
Hhex
Hhip
Hif3a
Hist1h1d
Hist1h2ae
Hist1h4i
Hmcn1
Hmga2-ps1
Hmha1
Homer2

Hrh1
Hs6st2
Hs6st3
Hspa12a
Htra4
Icos
Ifi30
Igf1
Igf2
Igfbp5
Igsf10
Igsf11
Igsf21
Ikzf4
Il11
Il13ra2
Il17ra
Il1rn
Il2rg
Il33
Il7r
Ildr2
Inf2
Inhbb
Inpp5d
Insc
Insl3
Irs3
Irx3
Itga2b
Itga4
Itga6
Itgam
Itgb2
Jam2
Kcnb1
Kcng4
Kcnj10
Kcnj2
Kcnk3
Kcnn1
Kcnn4
Kctd15
Kif19a
Kif1a
Kif5a
Kif5c
Kif9

Klhdc7a
Klhdc8a
Klk8
Klra2
Krt18
Krt80
Lacc1
Lamb3
Lamc3
Laptm5
Lbp
Lcn2
Lcn8
Lcp1
Lepr
Lfng
Lgals3
Lgi2
Lilrb4
Limch1
Lims2
Liph
Lmcd1
LOC100503676
Lox
Lpar6
Lrr1
Lrrc27
Lrrc4c
Lrrc66
Lrrn3
Lrtm1
Lsp1
Lst1
Ltbp2
Ly9
Lypd1
Lyplal1
Lyve1
Lyz1
Lyz2
M1ap
Mab21l3
Mafa
Mal2
Mamdc2
Map3k9
Map4k1

Mapk8ip2
Mast1
Mb
Mbp
Mcf2
Mcf2l
Mcoln2
Megf6
Met
Mfsd2a
Mfsd6
Mgam
Mgat4a
Mgst1
Mis18bp1
Mmd
Mmp12
Mmp13
Mmp19
Mmp27
Mmp8
Mpzl3
Mrgpre
Msi1
Mt3
Mybpc3
Mycl
Mycn
Myct1
Myh14
Mylk3
Myo1e
Myof
N28178
Naip5
Naip6
Naip7
Ncapg2
Nceh1
Ncf2
Nckap1l
Nckap5
Nek6
Ngef
Nhlrc4
Nipal3
Nlrc3
Nlrp10

Npl
Nr1h3
Nrap
Nrep
Nrn1
Nrp2
Nrros
Nrxn1
Nt5e
Ntrk2
Ntrk3
Nxpe4
Nxph3
Nynrin
Oas3
Ociad2
Odf3
Olr1
Onecut2
Oprd1
P2rx1
P2rx7
P2ry12
Pacsin1
Pak1
Papln
Parm1
Parvg
Pcbd1
Pcdh15
Pcdh20
Pcolce2
Pde1b
Pde2a
Pdgfb
Pdgfc
Pdk1
Pdpn
Perp
Pgap1
Phactr3
Pi15
Pid1
Piezo2
Pik3ap1
Pik3cb
Pik3cd
Pik3r5

Pik3r6
Pilra
Pitpnc1
Pkd1l2
Pla1a
Pla2g15
Pla2g4a
Pla2g7
Plagl1
Plat
Plau
Plaur
Plbd1
Plcg2
Plcxd2
Plek
Plekho1
Plxdc1
Plxna2
Pmaip1
Pparg
Ppargc1a
Ppef2
Ppfia4
Ppl
Ppm1j
Prkar1b
Prkcb
Prkch
Prss46
Prune2
Psat1
Psd4
Pstpip1
Ptgs1
Ptgs2
Ptk2b
Ptplad2
Ptpn22
Ptpn6
Ptprb
Ptpre
Ptprn
Ptprt
Ptprv
Pyhin1
Rab11fip1
Rab11fip4

Rab27a
Rab32
Rab38
Rac3
Rap1gap2
Rasa4
Rasgrf2
Rasgrp1
Rassf6
Rbm47
Rcan2
Reck
Reps2
Rftn1
Rgcc
Rgs16
Rgs19
Rgs2
Rgs7bp
Rhou
Ripk3
Rnf128
Rnf144b
Rnf157
Rnf180
Ropn1l
Rps6ka1
Rtn4rl1
Rufy4
Runx1
S100a16
S100a4
S100a6
S100a8
S1pr1
Saa3
Sall1
Samsn1
Scara3
Scgb3a1
Schip1
Scn3b
Scn8a
Sdc1
Sdk2
Sdpr
Sdsl
Sectm1a

Sele
Selp
Serinc2
Serpina1e
Serpina3h
Serpinb1c
Serpinb8
Serpinb9b
Sertad4
Sgip1
Sh2d2a
Sh3bgrl2
Sh3kbp1
Shc2
Shisa9
Shroom1
Ska1
Slamf9
Slc13a5
Slc14a1
Slc16a10
Slc16a12
Slc16a13
Slc18a1
Slc1a7
Slc22a12
Slc22a23
Slc22a4
Slc22a7
Slc26a10
Slc26a5
Slc2a3
Slc2a6
Slc38a1
Slc38a11
Slc47a2
Slc4a8
Slc7a11
Slc8a1
Slc8a3
Slc9a7
Slco2a1
Slfn10-ps
Slfn2
Slfn3
Slit3
Smpdl3b
Sned1

Snx10
Snx20
Soat1
Soat2
Sorbs2
Sorcs3
Sorl1
Sox17
Sox8
Sp5
Spag4
Spc24
Spc25
Spdl1
Specc1
Spire2
Spn
Spp1
Sprn
Sprr1a
Spsb4
Srcin1
Srgap1
Srgap3
Srgn
Srms
St14
Steap1
Steap3
Sulf2
Susd1
Svep1
Syce1
Syk
Syn1
Syngr1
Syngr3
Syt12
Tagap
Tarsl2
Tbc1d2
Tbc1d9
Tbx19
Tbxas1
Tdo2
Tdrkh
Tec
Tenm3

Tes
Tfec
Tfpi2
Tgfbr3
Ticam2
Ticrr
Timp1
Tlr6
Tlr7
Tm4sf1
Tm4sf19
Tm4sf4
Tmc3
Tmeff1
Tmem102
Tmem106a
Tmem108
Tmem117
Tmem132a
Tmem144
Tmem150c
Tmem151a
Tmem171
Tmem180
Tmem200a
Tmem202
Tmem26
Tmem37
Tmem8
Tmsb15b1
Tmsb15l
Tnf
Tnfrsf11a
Tnfrsf22
Tnfrsf23
Tnfrsf26
Tnfsf15
Tnfsf18
Tnip3
Tns4
Tox2
Tpd52
Trem2
Trib3
Tril
Trim16
Trpc4
Trpm2

Trpm6
Tspan13
Tspan2
Tspan33
Tspan5
Tspan8
Ttc7
Ttyh1
Tulp2
Uhrf1
Umps
Unc13a
Ung
Vav3
Vdr
Vgll3
Vill
Vldlr
Vnn1
Wbscr17
Wdr86
Wfdc3
Wfdc6a
Wisp2
Wnt2
Wscd1
Xylt1
Ydjc
Zcchc3
Zfhx2
Zfp423
Zfp612
